# Heterogeneous Photochemical Process Intensification: Crystallization-Induced Dynamic Resolution of Amines

**DOI:** 10.1021/acs.oprd.6c00152

**Published:** 2026-06-29

**Authors:** Jonathan M. Meinhardt, Jiang Wang, Yufei Wei, Daniel P. Walker, Robert R. Knowles

**Affiliations:** † Department of Chemistry, 6740 Princeton University, Princeton, New Jersey 08544, United States; ‡ Synthetic Molecule Design and Development, Lilly Research Laboratories, 1539 Eli Lilly and Company, Indianapolis, Indiana 46285, United States

**Keywords:** amines, crystallization, photochemistry, photocatalysis, heterogeneous reactions, dynamic resolution

## Abstract

The scale-up of solid-containing photochemical reactions is inherently challenging due to issues of light scattering and mass transport, which reduce productive light absorption and decrease reaction rates. However, reactive crystallizations are frequently employed as elements of process design, where *in situ* precipitation of products can drive equilibria or streamline purification operations. Herein we describe the implementation of a photoredox-driven crystallization-induced dynamic resolution of a racemic amine on multidecagram scale. This study features a recirculating photoreactor that continuously filters the reaction slurry containing the crystallized product and returns the clarified soluble phase for further irradiation and crystallization. By reducing crystal holdup and mitigating light attenuation in the optically dense reaction mixture, this configuration shortens reaction times and accelerates enantioenrichment compared to non-recirculating setups, enabling 1000-fold intensification of the original conditions reported in batch.

## Graphical abstract

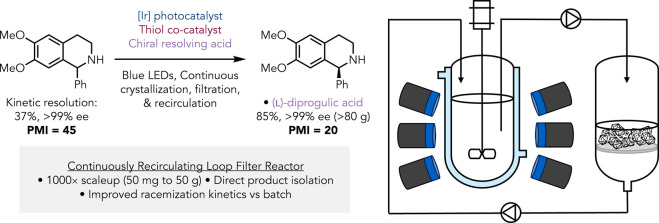

## Introduction

Crystallization-induced diastereomer transformations (CIDTs) are an attractive strategy for asymmetric synthesis, combining thermodynamically favorable crystallization equilibria with *in situ* racemization processes to precipitate the desired product stereoisomer from solution.^1^ Inspired by the growing body of CIDT literature, we recently disclosed a methodology for crystallization-induced dynamic resolution (CIDR) of racemic α-chiral amines using visible light-driven photoredox amine racemization and *in situ* diastereomeric crystallization with chiral resolving acids (Figure 1A).^2^ This strategy allows for efficient conversion of racemic amines into enantioenriched ammonium salts, providing an alternative to existing asymmetric amine syntheses and offering significantly improved yield efficiency compared to traditional kinetic resolution (KR).

**Figure 1:**
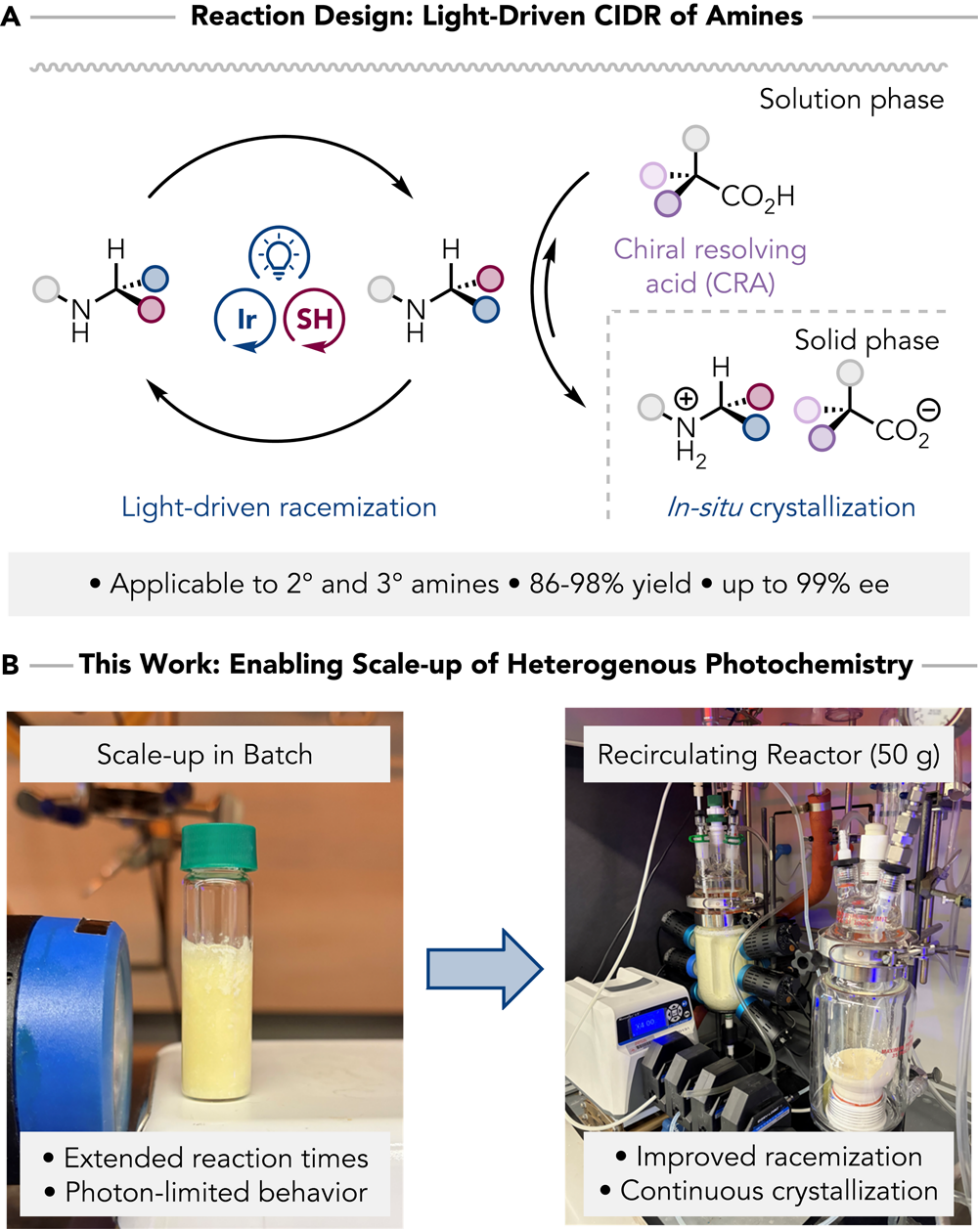
(A) Reaction design for light-driven CIDR. (B) Photochemical reaction scale-up enabled by a continuously recirculating filter loop reactor.

During the course of our original study, we recognized that practical use of the CIDR would involve implementation on preparative scales beyond those typically encountered in academic methodology development. However, the CIDR is both heterogeneous and photon-driven, design aspects which complicate potential scale-up efforts.^3^ Although numerous reports describing intensification of solid-containing photochemical reactions have been disclosed,^4,5^ heterogeneity in these systems is often due to insoluble starting materials, which may gradually enter solution over the course of a reaction. In contrast, the CIDR necessarily produces an insoluble salt that crystallizes directly from the reaction mixture. With increasing reaction scale, the large amount of precipitated salt product presents increasingly significant issues of mass transport and reduced light transmission through the reaction medium.^3a,6^ Aware of these challenges, we sought to develop an approach that would enable CIDR on scales bridging laboratory experiments and larger preparative campaigns.

Herein we present the implementation of light-driven CIDR on multidecagram scale using a continuously recirculating loop reactor design (Figure 1B).^7^ In addition to irradiation and crystallization conducted in a conventional stirred vessel, this design incorporates recirculation of the reaction slurry through an ancillary filter loop, facilitating continuous product removal and slurry dilution. We demonstrate that this reactor configuration allows for 1000× scale-up of the original CIDR developed in batch, enables improved reactor productivity, and can be used to directly isolate up to 85 g of diastereomeric salt product in high yield and selectivity. We hope that the concepts explored in this study will inform future implementations of solid-containing photochemical reactions and improve their translation into production settings.

## Results and Discussion

### Limitations of Reaction in Batch

Our scale-up efforts focused on the dynamic resolution of *rac*-6,7-dimethoxy-1-phenyl-1,2,3,4-tetrahydroisoquinoline (**1**) using l-diprogulic acid monohydrate (**2**) as the resolving acid. Data describing the yield and selectivity of the analogous kinetic resolution of *rac*-**1** are presented in Table 1. In the CIDR, amine racemization is mediated by [Ir­(dF­(Me)­ppy)_2_(dtbbpy)]­PF_6_ photocatalyst, blue-light irradiation, and 2,4,6-triisopropylbenzenethiol (TRIP–SH) cocatalyst. Under optimized small-scale conditions (50 mg scale), the CIDR reaches an enantioenrichment of 94% ee with near-quantitative yield after 12 h of irradiation. Notably, the dynamic resolution is also selective across a range of concentrations (Table 2, entries 1–4) and can accommodate iridium photocatalyst loadings as low as 0.25 mol % (entries 5 and 6), factors which were identified as desirable from a process intensification standpoint. Upon completion of the CIDR, the diastereomeric salt product **3** forms a flowing suspension at reaction concentrations ≤ 0.2 M.

**Table 1:**
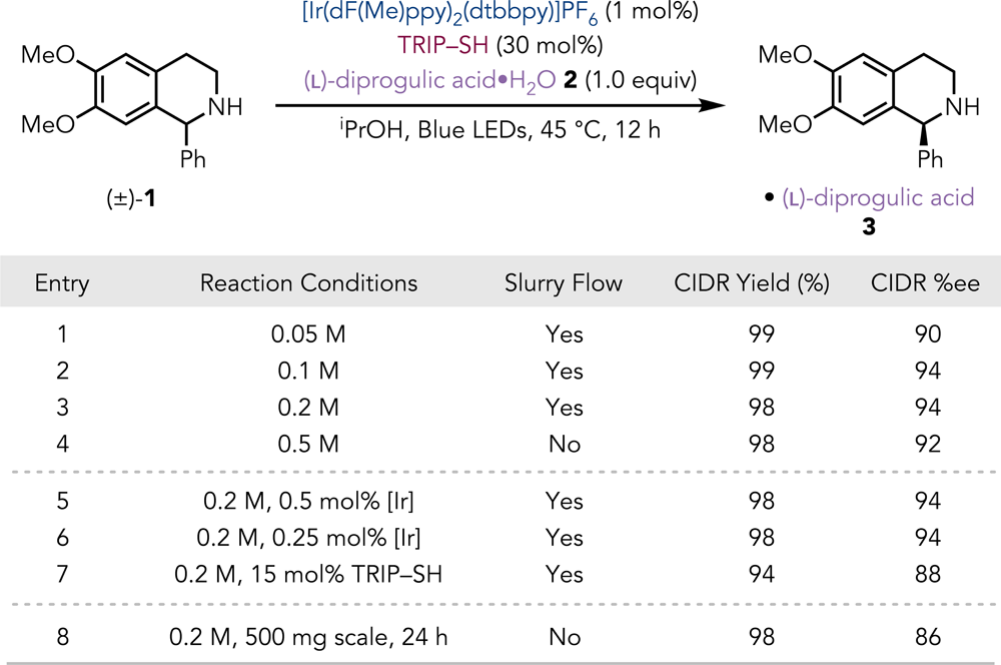
Kinetic Resolution of *rac*-**1** ^a^

a Reactions were conducted on 500 mg scale. Yield and selectivity data are for isolated diastereomeric salt. Slurry flow = visually unimpeded movement of the reaction mixture under magnetic stirring (800 rpm).

**Table 2:**
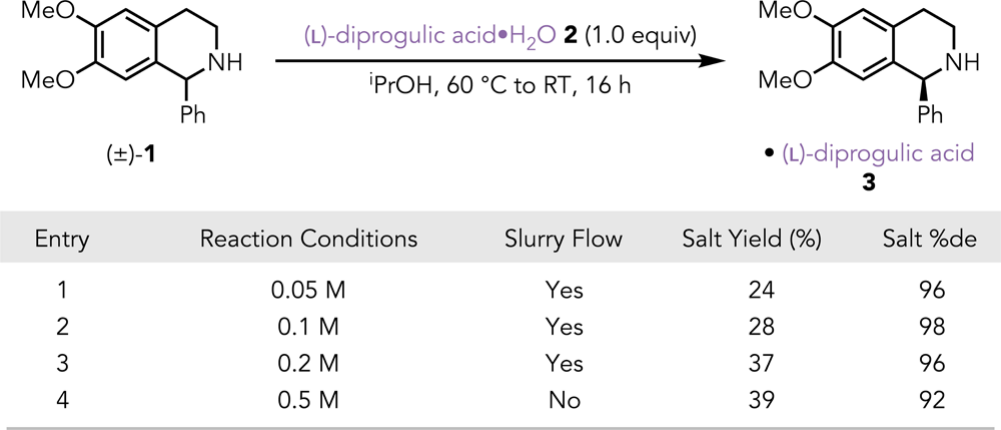
Vial-Scale Reaction Optimization^a^

a Reactions were performed on 0.2 mmol (50 mg) scale unless otherwise noted. CIDR yields and selectivity correspond to the freebase amine from the entire reaction mixture (both the soluble and insoluble phases). Yields were determined by ^1^H NMR spectroscopic analysis of the crude reaction mixture against 1,3,5-trimethoxybenzene internal standard.

Initial 10× scale-up of the reaction to 500 mg in batch required increased reaction times and gave lower overall selectivity (98%, 86% ee after 24 h; Table 2, entry 8). Moreover, reactions conducted at 500 mg scale displayed a significant decrease in the rate of enantioenrichment in the solution phase (Figure 2A, dark red trace) through the loss of the asymptotic evolution of solution ee seen in 50 mg scale experiments (dark blue trace). Instead, the slow linear buildup of solution ee observed at 500 mg scale is diagnostic of a kinetic regime wherein the rate of dynamic resolution is governed by photon flux into the reactor rather than the intrinsic kinetics of racemization and crystallization. This change in kinetic behavior likely reflects a reduced irradiated surface-area-to-volume ratio, decreased mixing efficiency within the slurry, or increasingly significant light scattering caused by the insoluble salt product **3**.

**Figure 2:**
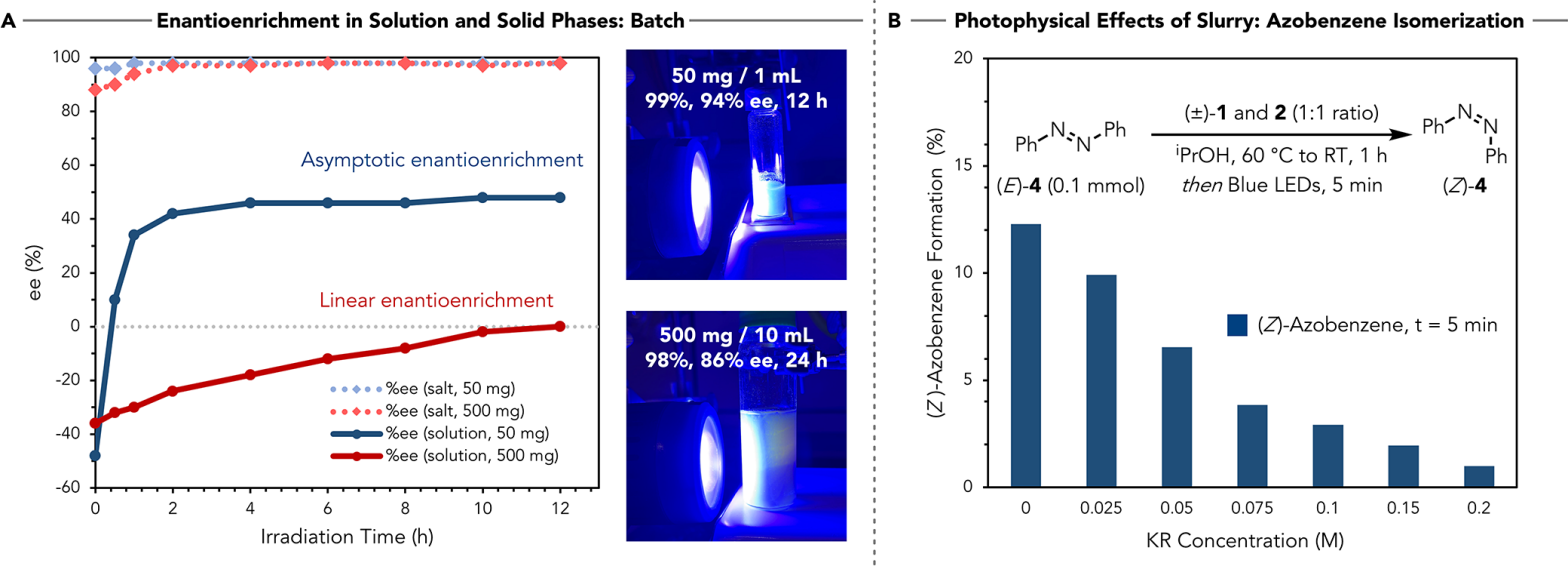
(A) Solution-phase amine racemization kinetics for 50 mg- and 500 mg-scale batch reactions. (B) Effects of increasing slurry concentration on the light-induced isomerization of (*E*)-azobenzene. KR concentration = initial concentration of *rac*-**1** and **2**.

To better describe the impact of the reaction slurry on light distribution within the reaction mixture, we conducted experiments measuring the isomerization yield of (*E*)-azobenzene (**4**) under blue-light irradiation at various slurry concentrations in the absence of iridium and thiol catalysts (Figure 2B).^8^ These data reveal a pronounced decrease in the extent of photochemical *E* to *Z* isomerization of **4** with increasing slurry concentration. In light of these results, we anticipated that further scale-up of the CIDR in a simple batch configuration would be untenable. We thus pivoted our optimization efforts toward reactor engineering strategies to mitigate issues of photon delivery and mass transfer encountered in batch moving to larger reaction scales.

### Transition to a Recirculating Reactor

Cognizant of the poor batch scalability of the CIDR, we sought to devise an approach that would improve the efficiency of light-driven racemization required for dynamic resolution. Inspired by continuous crystallization strategies such as mixed-suspension mixed-product-removal (MSMPR) crystallization,^9^ we envisioned that a reactor which facilitated continuous removal of the insoluble product **3** would provide a tenable avenue toward scale-up. However, the need to resubject remaining soluble amine **1** to further irradiation and crystallization to achieve high yield efficiency in the CIDR prevents the implementation of a truly continuous MSMPR. Accordingly, we designed a recirculating filter loop that would (1) continuously remove the insoluble salt product from the irradiation vessel and (2) return the clarified mother liquor to the irradiation vessel (Figure 3A). The recirculating reactor consists of a stirred vessel surrounded by an array of 456 nm LED lamps for simultaneous irradiation and crystallization, with a separate filter assembly for product collection and slurry filtration. As designed, the continuously crystallizing reaction mixture would be gradually diluted over the course of irradiation, transforming the soluble racemic amine into an isolated enantioenriched salt product. Alternative reactor configurations such as continuous stirred tank reactor (CSTR) cascades^10^ were also considered as viable approaches but were not tested due to equipment constraints. Although commonly employed for the scale-up of photochemical reactions,^11^ plug-flow reactors (PFRs) were deemed unsuitable for this study due to potential fouling of tubing due to crystal holdup and limited solid–liquid mixing capability.^12^

**Figure 3:**
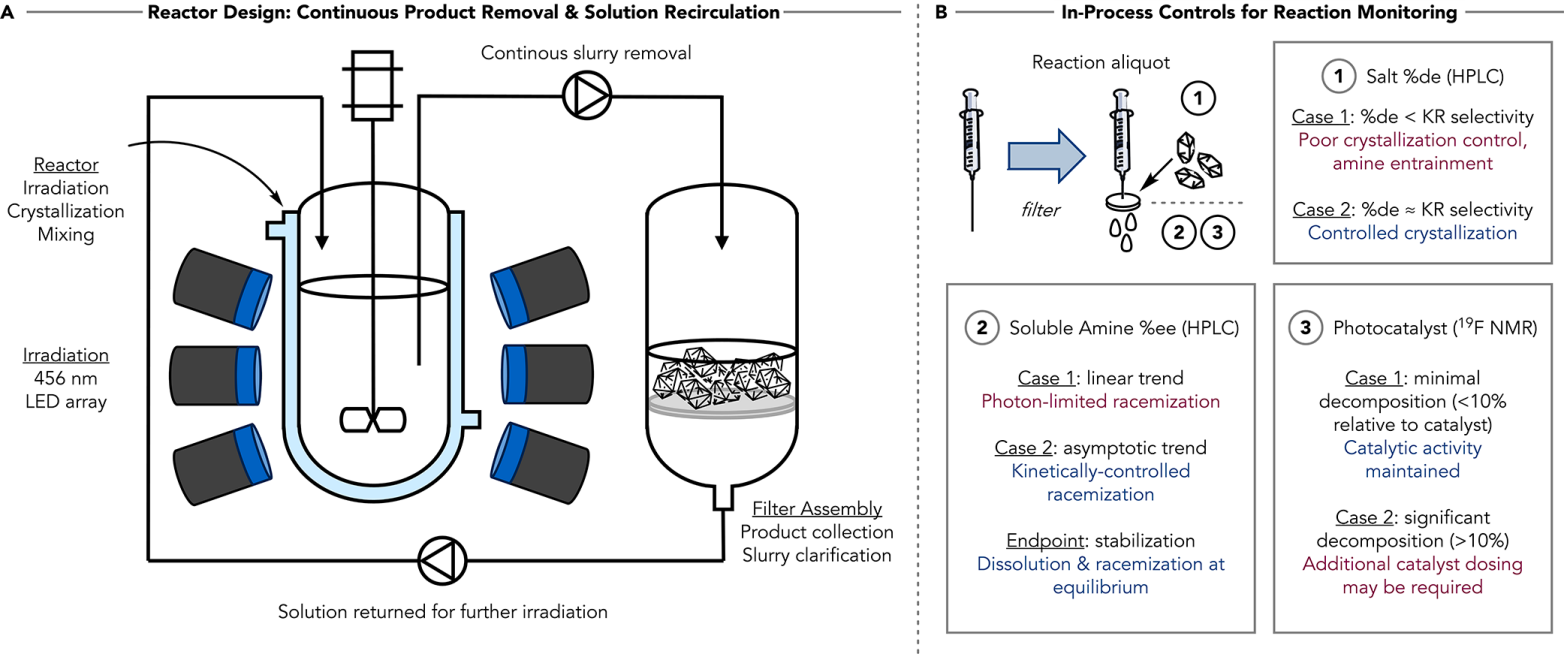
(A) Schematic of the recirculating loop filter reactor. (B) In-process controls used to assess reaction progress during CIDR operation.

### In-Process Controls (IPCs) for Recirculating Reactor

Because of the multiphasic nature of the CIDR, developing a detailed model for kinetic analysis of reaction progress is complex. Rates of crystallization, dissolution, and substrate racemization are intrinsically coupled to crystal holdup, which simultaneously influences phase-transfer kinetics and photon distribution through the reaction medium. Rather than attempt to explicitly resolve these interactions, we sought to develop controls that would allow for real-time identification of favorable operating regimes without reliance on detailed kinetic analysis.

We thus established the following IPCs to monitor reactions conducted in the recirculating setup (Figure 3B). During sampling of the batch reaction at both 50 and 500 mg scale, the % de of the precipitated salt product **3** remained essentially constant throughout the reaction near the kinetic resolution limit (ca. 96% de). In contrast, the % ee of the fraction of amine in the solution phase differed markedly, showing fast, asymptotic enantioenrichment at 50 mg scale and slow, linear enantioenrichment at 500 mg scale (Figure 2A). These distinct solution-phase kinetic profiles would provide a practical diagnostic for the kinetic regime operating in the recirculating reactor, with plateauing asymptotic enantioenrichment indicating a productive departure from photon-limited amine racemization kinetics. Accordingly, solution-phase enantioenrichment was monitored during scale-up experiments via HPLC analysis of filtered reaction aliquots collected over time. Photocatalyst decomposition was also assessed in parallel by ^19^F NMR spectroscopy during the course of scale-up runs to monitor potential catalyst deactivation.^13^

The rate of solid removal was also considered as a key optimization parameter in the recirculating reactor, impacting both the residence time distribution of crystallization and the extent of supersaturation in the reactor vessel.^14^ If solids were removed too slowly, the CIDR would not fully capture potential racemization rate acceleration enabled by slurry dilution. Conversely, rapid removal of the crystallized product at a rate exceeding optimal crystal growth could result in a nucleation-dominated crystallization regime, producing fines or creating losses in resolution selectivity or product yield.^15^ Conscious of the importance of a balanced solid removal rate, an empirical approach was taken to optimize the residence time of the racemizing slurry in the reactor.^16^

### Proof-of-Concept Demonstrations (10–25 g CIDR)

Initial design studies focused on handling the slurry produced from kinetic resolution of *rac*-**1** using a multichannel peristaltic pump. No issues with tubing fouling (1/8” I.D. tubing) were encountered at slurry concentrations of 0.2 M, and the suction created by the pump was sufficient to drive filtration through filter cakes of product **3**. Moreover, the salt product **3** remains suspended in the pump tubing for extended periods of time (>1 h), allowing for pumping rates as low as 0.1 mL/min without appreciable sedimentation. In the absence of obvious technical issues with slurry transport, we targeted an initial multigram demonstration of the recirculating reactor using 10 g of racemic amine (Figure 4A). This reaction was conducted in a 250 mL round-bottom flask configured for magnetic stirring with an iridium photocatalyst loading of 0.5 mol % at a reaction concentration of 0.2 M (see the Supporting Information for detailed reactor description). Following 10 h of irradiation with a slurry removal rate of 1 mL/min (idealized τ_res_ = 3 h), crude **3** was isolated in 93.3% yield with 94.6% ee after drying of the collected filter cake. Subsequent ^i^PrOH reslurry of the crude product afforded 17.2 g of product **3** (85.3% overall yield) enriched to 99.4% ee. In addition to the high yield and selectivity obtained at 10 g scale, completion of the reaction in approximately 10 h further bolstered our process intensification efforts, providing a reaction time scale comparable to that of the optimized small-scale CIDR.

**Figure 4:**
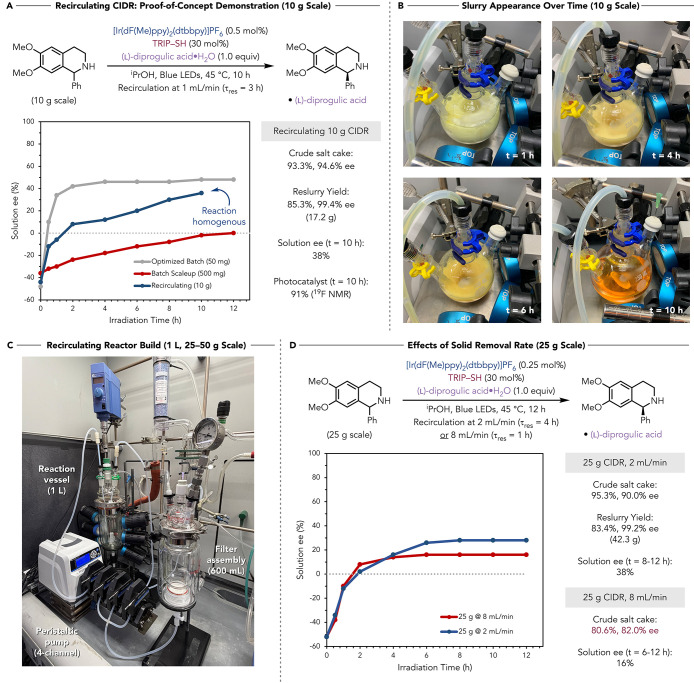
(A) Recovery of solution enantioenrichment kinetics during initial 10 g demonstration in recirculating reactor setup. Yields are for isolated diastereomeric salt **3**. (B) Appearance of 10 g reaction showing slurry dilution over time. (C) Transition to 1 L kettle reactor with overhead stirring for 25–50 g experiments. (D) Yield and solution % ee data for 25 g scale reactions with different solid removal rates.

While an encouraging proof-of-concept validation of the recirculating reactor design, several key observations were made during the 10 g experiment. First, irradiation was stopped after 10 h due to the formation of a homogeneous solution in the reaction vessel, forcing an end point due to a lack of further crystallization. Images of the reaction vessel at various time points are shown in Figure 4B, depicting gradual dilution of the reaction slurry and solution homogeneity at completion. Second, in-process monitoring of solution enantioenrichment indicated recovery from the slow, linear regime encountered in 500 mg batch reactions, with the solution becoming enantioenriched in the resolving amine stereoisomer after ca. 2 h of reaction time (Figure 4A).^17^ Compared to the solution ee profile of the optimized 50 mg batch reaction (stabilizing at 48% ee), the 10 g recirculating reaction reached a lower solution enantioenrichment at completion (38% ee), reflective of product removal and a reduced extent of salt redissolution. Together with yield and selectivity data for isolated product **3**, this kinetic profile at 10 g scale suggested that the recirculating reactor enabled efficient scale-up of the CIDR. Finally, ^19^F NMR spectroscopic analysis revealed 9% decomposition of the iridium photocatalyst at completion, defining an acceptable level of catalyst degradation during the reaction.

With the viability of the recirculating reactor established, we next addressed remaining barriers toward further scale-up of the CIDR. To better reflect production-relevant operating conditions, subsequent demonstrations were conducted in a 1 L jacketed glass reactor equipped with overhead stirring. Irradiation in this setup was provided by an array of eight 34 W LED lamps (Figure 4C). Although no stirring issues were observed for 10 g scale reactions, a transition away from magnetic stirring is not only more representative of production-scale reactors but would also clarify if mechanically induced crystal attrition was necessary or beneficial for CIDR performance.^18,19^

The updated reactor configuration was first tested at 25 g scale using a further reduced photocatalyst loading of 0.25 mol %. The pumping rate was set at 2 mL/min, providing an idealized τ_res_ of 3.8 h. After 12 h of irradiation, the reaction had reached a stable solution enantioenrichment of 28% ee (Figure 4D, blue trace). The crude salt product was isolated in 95.3% yield with 90.0% ee and subsequently polished to 83.4% overall yield with 99.2% ee (42.3 g) via ^i^PrOH reslurry. By the end of the reaction, photocatalyst decomposition had reached 8% by ^19^F NMR spectroscopic analysis. Furthermore, solution enantioenrichment evolved similarly to the previous 10 g recirculating reaction at 25 g scale. In an attempt to increase the space-time yield of the reactor, a second 25 g reaction was performed using an increased pumping rate of 8 mL/min (idealized τ_res_ = 1 h) (Figure 4D, red trace). However, this reaction yielded a diminished crude yield of 80.6% with lower enantiopurity in the product filter cake (82.0% ee) after washing. Losses in salt yield were attributed to a reduced extent of crystal growth in the crystallizer due to a shorter τ_res_, and the reduced enantiopurity of the accumulated filter cake was attributed to increased entrainment of undesired amine stereoisomer due to poor cake wetting during recirculation.^20^ Although unsuccessful in producing an efficient CIDR, this result underscores the importance of a balanced solid removal rate to maintain steady racemization and maximize crystal growth.

### Proof-of-Process Demonstration (50 g CIDR)

With an understanding of the CIDR in the recirculating reactor established, we directly translated the conditions used for the successful 25 g reaction to 50 g scale (Figure 5A). Maintaining a τ_res_ of 3.8 h, CIDR conducted at 50 g scale reached a stable solution enantioenrichment of 26% ee after 14 h of irradiation, again asymptotically converging to a stable plateau during the course of the reaction. Product accumulation proceeded steadily in the filter assembly (Figure 5B), and gradual dilution of the reaction slurry was visually evident over the course of the reaction (Figure 5C).^21^ After an EtOAc wash of the collected filter cake and subsequent drying, crude **3** was obtained in 93.7% with 89.6% ee (ca. 94.7 g). Following a final reslurry from ^i^PrOH, **3** was obtained in 85.4% overall yield with 99.7% ee (86.4 g). We were pleased to observe that significant extension of the reaction time between 25 and 50 g scale was not required to achieve a high-yielding dynamic resolution using the 1 L reactor setup. As implemented above, the CIDR operates with a process mass intensity (PMI) of 20, representing a meaningful improvement in efficiency compared to the analogous kinetic resolution (PMI = 45).

**Figure 5:**
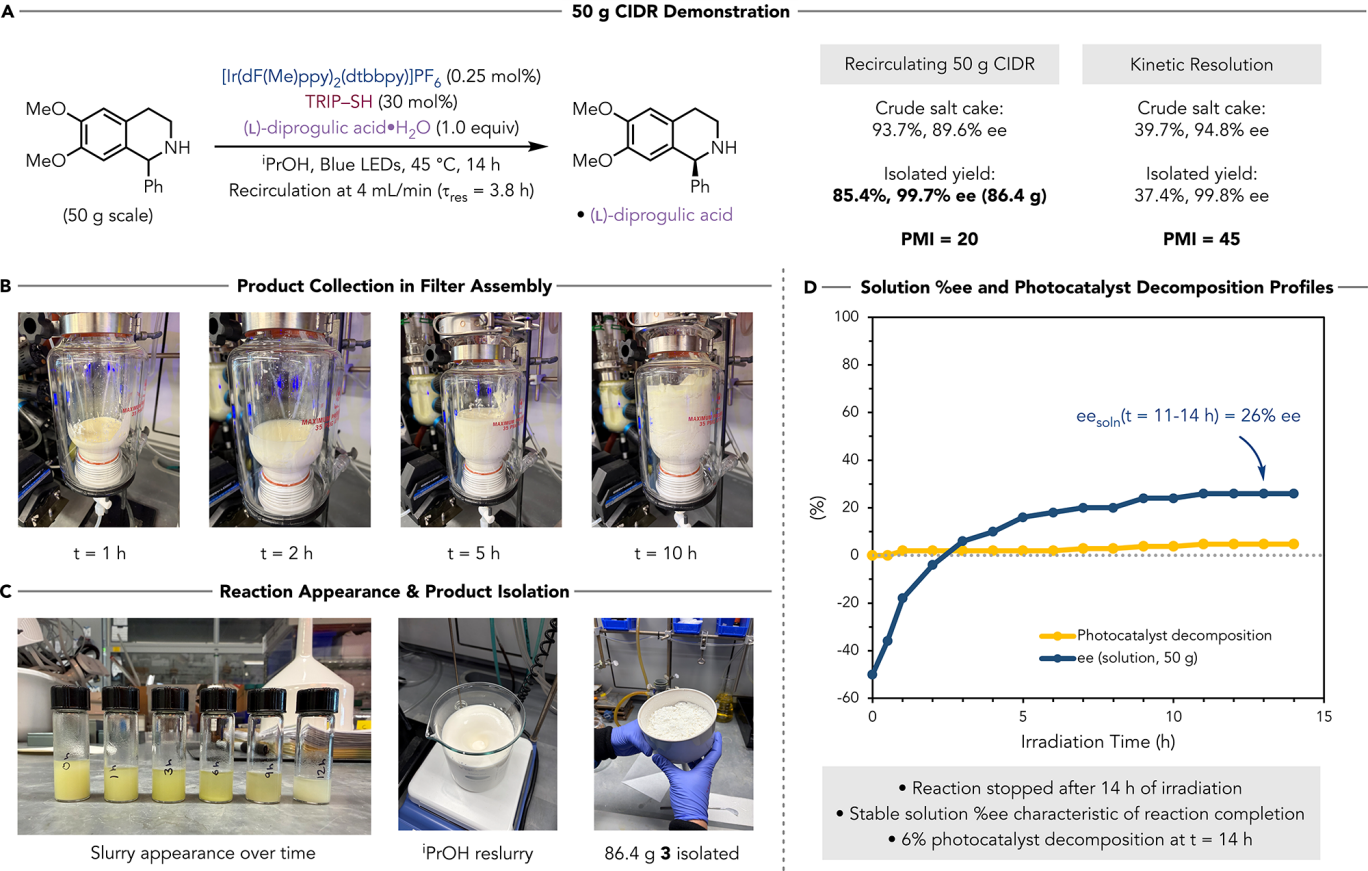
(A) Recirculating CIDR at 50 g scale. (B) Product accumulation in ancillary filter assembly. (C) Dilution of reaction slurry over time and post-reaction processing operations. (D) 50 g reaction timecourse including solution enantioenrichment behavior and photocatalyst decomposition (monitored by ^19^F NMR spectroscopic analysis).

Across the reaction scales studied during this work, differences in vessel geometry and mixing configuration complicate direct comparisons of light delivery on the basis of nominal LED power alone (Table 3). Given the high optical density of the reaction slurry, these reactions are expected to operate in a surface-limited regime with photon absorption occurring within a shallow penetration depth near the irradiation vessel walls.^6^ Under these conditions, slurry concentration and mixing behavior further influence the distribution of reaction components within the illuminated zone. Accordingly, light delivery during the course of the reaction is more appropriately considered as a function of the illuminated surface area rather than total solution volume. In the present study, all reactors employed externally mounted LED arrays positioned around each vessel. Alternative configurations, such as immersion well light sources,^4a^ may enhance the efficiency of light distribution and could prove advantageous at scales beyond those examined in this study.

**Table 3:**
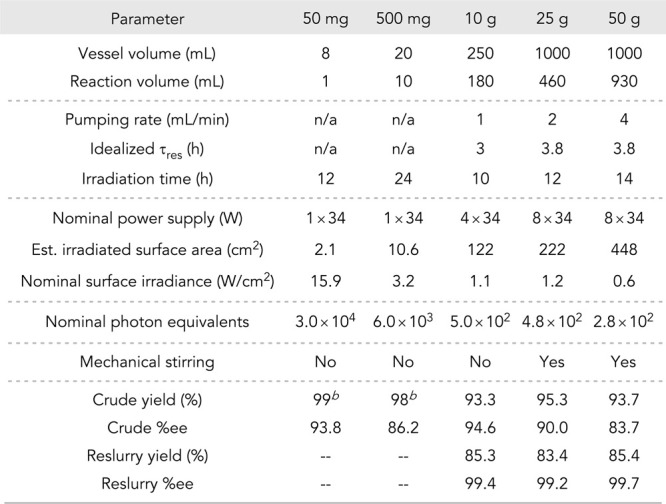
Reactor Parameters from 50 mg to 50 g Scale^a^

a Power supply calculations assume full delivery of nominal lamp wattage to the reactor vessel and are thus overestimated.

b Yields were determined by ^1^H NMR spectroscopic analysis of the crude reaction mixture against 1,3,5-trimethoxybenzene internal standard.

## Conclusions

In summary, this study demonstrates the scale-up of a light-driven reactive crystallization enabled by a continuous product removal strategy. Integration of a recirculating filter loop with a stirred irradiation/crystallization vessel enabled continuous dilution of the reaction slurry, alleviating issues related to the inefficient light delivery encountered in small-scale batch reactions. The recirculating reactor enables recovery of efficient solution-phase racemization kinetics and increases the overall productivity of the CIDR, shown through a series of decagram-scale reaction demonstrations on up to a 50 g scale. More broadly, this work provides a case study for the process intensification of solid-containing photochemical transformations, applied here for efficient production of enantioenriched chiral amines using a crystallization-induced dynamic resolution.

## Experimental Information

### General

All commercial reagents and solvents were used as received. Anhydrous solvents were used directly from Sigma-Aldrich Sure/Seal containers. ^1^H, ^13^C, and ^19^F NMR spectra were recorded on Bruker 500 instruments (500, 126, and 471 MHz, respectively). ^1^H and ^13^C NMR spectra are internally referenced to residual solvent (δ = 7.26 and 77.16 ppm for CDCl_3_). ^19^F NMR spectra are referenced to fluorobenzene as an internal standard (PhF referenced at δ = −113.15 ppm). NMR data are reported as follows: chemical shift (multiplicity, coupling constant(s), integration). Chemical shifts (δ) are in parts per million. Multiplicities are denoted as follows: s = singlet, d = doublet, t = triplet, q = quartet, p = pentet, sext = sextet, hept = septet, m = multiplet, brs = broad singlet, brd = broad doublet). Coupling constants (*J*) are given in hertz. No special nomenclature is used for equivalent carbons. IR spectra were recorded on a Thermo Scientific Nicolet 6700 FT-IR spectrometer and are reported in terms of wavenumber (cm^–1^). High-resolution mass spectra were obtained at Princeton University mass spectrometry facilities using an Agilent 6230 TOF LC/MS. Analytical HPLC analyses were performed on Agilent 1260 series HPLC instruments equipped with ChiralCel OD-H (5 μm particle size, 4.6 mm × 250 mm) columns from Daicel Chemical Industries. Optical rotations were measured using a Jasco P-2000 polarimeter and were obtained at 589 nm in a cell with a path length of 100 mm. Concentration values (*c*) for optical rotation values are reported in g/100 mL. Particle size distributions (PSDs) were measured via laser diffraction using a Malvern Mastersizer 3000 equipped with an MV liquid disperser. Electron microscopy (EM) analysis was conducted using an FEI Teneo electron microscope with an Everhart–Thornley secondary electron detector.

#### Safety Note

The experiments described herein involve the use of high-intensity light sources (456 nm). All photochemical reactions were conducted in an enclosed, shielded reactor setup to minimize operator exposure to irradiation. Appropriate personal protective equipment, including blue-light-blocking safety glasses, lab coats, and gloves, was worn during reactor operation. All experiments were performed in well-ventilated fume cabinets.

### Reactor Design

Descriptions of all reactor setups are provided in the Supporting Information. The recirculating reactor used between 25 and 50 g scale consists of a 1 L jacketed glass reactor fitted with an overhead stir shaft (irradiation/crystallization vessel), a 600 mL jacketed glass filter reactor (filter/product collector vessel), a four-channel peristaltic pump, and an array of 34 W 456 nm LED lamps (Kessil). Reactor jacket temperature was regulated by means of recirculating independently controlled heat transfer fluid. All connections between reactor vessels were plumbed using 1/4″ O.D., 1/8″ I.D. PTFE or silicone tubing with PTFE or PVDF compression unions between tubing types. Connections to reactor ports were made using appropriate threaded or ground-glass adapters. Slurry removal was facilitated using an adjustable PTFE dip tube inserted into a port in the irradiation/crystallization vessel. After filtration, the clarified slurry was returned to the reactor vessel for further irradiation via a second line from the bottom of the filter vessel to an inlet port on the irradiation vessel. All components used to assemble the reactor were obtained from commercial suppliers.

### Synthesis of *rac*-6,7-Dimethoxy-1-phenyl­tetrahydroisoquinoline (*rac*-**1**)

To a 1 L round-bottom flask equipped with a magnetic stir bar was added 3,4-dimethoxyphenethylamine (50.000 g, 275.87 mmol, 1.0000 equiv) and benzaldehyde (29.268 g, 275.87 mmol, 1.0000 equiv). The mixture was dissolved in methanol (275 mL, 1.0 M solution) and stirred for 16 h at room temperature with stirring at 800 rpm. The mixture was concentrated under reduced pressure to afford the corresponding imine intermediate which was carried forward without further purification. To the intermediate residue in a 1 L round-bottom flask was carefully added trifluoroacetic acid (100 mL, 2.8 M solution). The mixture was heated to 85 °C for 3 h, cooled to room temperature, then cooled to 0 °C, and diluted with distilled water (200 mL). The resulting solution was basified with 15 wt % aqueous NaOH solution (400 mL), and the mixture was extracted with CH_2_Cl_2_ (3 × 100 mL), dried over Na_2_SO_4_, and concentrated under reduced pressure. The residue was then suspended in 100 mL Et_2_O and cooled at −10 °C for 2 h. The precipitated solids were filtered and washed with Et_2_O (3 × 100 mL) to afford the product **1** as a coarse white solid in 78.4% yield (59.078 g). Characterization data were consistent with literature reports.^22^

### Representative Procedure for Preparation of Salt **3** via Kinetic Resolution of *rac*-**1**

#### Resolution

To a 500 mL round-bottom flask equipped with a magnetic stir bar and a reflux condenser were charged racemic amine **1** (10.000 g, 37.128 mmol, 1.000 equiv) and l-diprogulic acid monohydrate (**2**) (10.852 g, 37.128 mmol, 1.000 equiv). ^i^PrOH (180 mL, 0.2 M solution) was then added to the reactor vessel, and the mixture was heated to 80 °C for 1 h with stirring at 1000 rpm. The mixture was then allowed to equilibrate to room temperature over the course of 12 h and filtered. The filter cake was washed with EtOAc (3 × 50 mL) and dried under air flow for 3 h, affording crude salt **3** in 39.7% yield with 94.8% ee (8.006 g).

#### Reslurry

To the crude **3** obtained from the initial resolution (ca. 8.0 g) was added ^i^PrOH (50 mL, 0.3 M solution). The mixture was stirred (1000 rpm) and heated to 80 °C for 1 h and then allowed to equilibrate to room temperature over the course of 4 h. The mixture was then filtered and dried under air flow for 3 h to afford **3** as a white solid in 37.4% overall yield with 99.8% ee (7.546 g).

### Representative Procedure for Recirculating Dynamic Resolution (50 g Scale)

#### Reaction

To a 1 L baffled jacketed reactor (R1) equipped for recirculation through a 600 mL filter reactor (R2) were charged racemic amine **1** (50.000 g, 185.64 mmol, 1.0000 equiv), acid **2** (54.258 g, 185.64 mmol, 1.0000 equiv), 2,4,6-triisopropylbenzenethiol (13.166 g, 30 mol %), and [Ir­(dF­(Me)­ppy)_2_(dtbbpy)]­PF_6_ (0.487 g, 0.25 mol %). The entire reactor assembly was purged with positive nitrogen pressure for 30 min. Separately, degassed anhydrous ^i^PrOH (930 mL, 0.2 M solution) was heated to 80 °C. The heated solvent was then cannulated into the reactor vessel, and the mixture was stirred at 1000 rpm for 30 min until an internal temperature of 45 °C was reached. Circulation of the reactor jacket fluid (45 °C) was started, and the reaction mixture was stirred for an additional 30 min. Irradiation (8 × 34 W Kessil PR160L-456nm blue LED lamps) and continuous recirculation (4 mL/min) were then initiated simultaneously with stirring maintained at 1000 rpm. For the first 3 h of solid removal, additional ^i^PrOH (3 × 50 mL) was delivered to R2 to maintain a liquid layer above the filter cake. Slurry removal was monitored, with aliquot removal through a reactor port at specified intervals. Upon completion, the remaining slurry in R1 was transferred to R2. The collected filter cake was washed with EtOAc (3 × 100 mL) and dried to afford the crude product **3** as a white solid in 93.7% yield with 89.6% ee (94.744 g). The crude product was determined to have a PSD of *d*_10_ = 1.8 μm, *d*_50_ = 6.9 μm, and *d*_90_ = 33.3 μm.

#### Reslurry

To the crude **3** obtained from the reaction (ca. 94.7 g) was added ^i^PrOH (580 mL, 0.3 M solution). The mixture was stirred (1000 rpm) and heated to 80 °C for 1 h and then allowed to equilibrate to room temperature over the course of 4 h. The slurry was then filtered and dried to afford the product **3** as a white solid in 85.4% overall yield with 99.7% ee (86.404 g). The reslurried product was determined to have a PSD of *d*_10_ = 4.3 μm, *d*_50_ = 490 μm, and *d*_90_ = 1368 μm. ^1^H NMR (500 MHz, CDCl_3_) δ: 9.09 (brs, 1H), 7.43–7.36 (m, 2H), 7.34–7.27 (m, 3H), 6.58 (s, 1H), 6.15 (s, 1H), 5.43 (s, 1H), 4.75 (s, 1H), 4.19 (d, *J* = 2.4 Hz, 1H), 4.14–3.95 (m, 2H), 3.85 (s, 3H), 3.82 (d, *J* = 13.3 Hz, 1H), 3.59 (s, 3H), 3.19 (ddd, *J* = 15.7, 9.0, 5.9 Hz, 1H), 3.13–2.98 (m, 2H), 2.73 (dt, *J* = 16.3, 4.7 Hz, 1H), 1.48 (s, 3H), 1.39 (s, 3H), 1.38 (s, 3H), 1.29 (s, 3H). ^13^C NMR (126 MHz, CDCl_3_) δ: 170.78, 148.42, 147.69, 139.32, 130.27, 128.81, 128.60, 126.26, 125.86, 112.46, 112.32, 111.20, 110.89, 97.59, 87.09, 73.65, 72.97, 60.14, 59.34, 56.02, 56.00, 40.22, 28.95, 27.27, 26.27, 26.18, 18.98. IR (neat): 2989, 2937, 2833, 1611, 1515, 1456, 1405, 1373, 1312, 1258, 1235, 1219, 1184, 1109, 1013, 1066, 874, 858, 835, 702, 529. HRMS (ESI): *m*/*z* calcd for C_17_H_20_NO_2_^+^ [M + H^+^] 270.14885, found 270.14865 (0.74 ppm); *m*/*z* calcd for C_12_H_17_O_7_^–^ [M^–^] 273.09798, found 273.09793 (0.18 ppm). [α]_D_ – 6.5 (*c* = 0.10, CHCl_3_).

## Supplementary Material



## Notes

Experimental details, solid-state characterization data, NMR spectra, and HPLC traces (PDF)
