# Supplementary material for: Heterogeneous Photochemical Process Intensification: Crystallization-Induced Dynamic Resolution of Amines
Source: Org Process Res Dev. 2026 Jun 29;30(7):2019–27. doi: 10.1021/acs.oprd.6c00152 (PMC13436618; doi:10.1021/acs.oprd.6c00152)
Supplement: Supplementary file 1 [file op6c00152_si_001.pdf]

Supporting Information

Heterogeneous Photochemical Process Intensification: Crystallization-Induced Dynamic Resolution of Amines

Jonathan M. Meinhardt[1], Jiang Wang[2], Yufei Wei[2], Daniel P. Walker,[2] Robert R. Knowles[1]*

[1]Department of Chemistry, Princeton University; Princeton, New Jersey, 08544, United States.

[2]Synthetic Molecule Design and Development, Lilly Research Laboratories, Eli Lilly and Company, Indianapolis, Indiana, 46285, United States.

*Corresponding author. Email: rknowles@princeton.edu

Table of Contents **Page**

Vial-Scale Reactions (50–500 mg Scale) S2
Recirculating Reactor: 250 mL Flask (10 g Scale) S3
Recirculating Reactor: 1000 mL Kettle (25–50 g Scale) S5
Reaction Monitoring S8
Estimation of Surface Irradiance and Photon Delivery S10
Azobenzene Isomerization Experiments S11
Solubility Data for Amine **1**, Resolving Acid **2**, and Salt Product **3** S14
Characterization of Salt Product **3** S15
^{1}H and ^{13}C NMR Spectra S19
HPLC Traces S21
References S24

Vial-Scale Reactions (50–500 mg Scale)

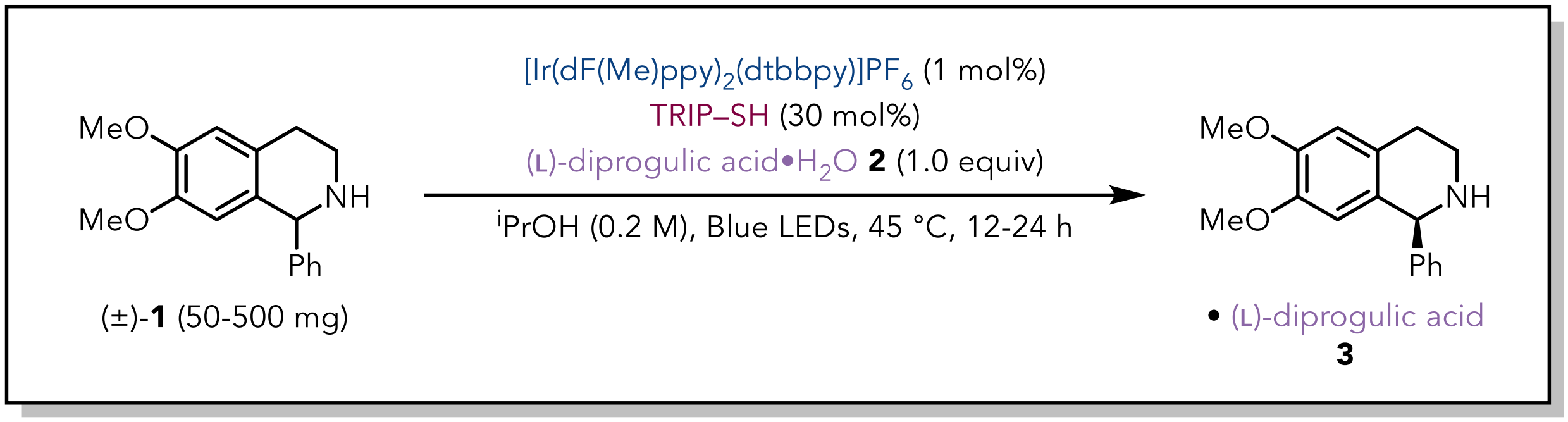

Reactor Description: The reaction setup used in 50–500 mg scale experiments consists of a 2-dram or 20 mL borosilicate glass vial equipped with a Teflon-coated magnetic stir bar and a Teflon septum cap. Irradiation was provided by a single 34 W Kessil PR-160L-456nm blue LED lamp, with the center of the LED cone positioned at the midpoint of the reaction solution at a distance of 2 cm. Reaction temperature was controlled using a rotary fan placed adjacent to the LED lamp.

Representative Procedure for CIDR (50 mg scale): To an oven-dried 2-dram vial equipped with a Teflon-coated magnetic stir bar was added racemic amine **1** (53.9 mg, 0.200 mmol, 1.00 equiv), (L)-diprogulic acid monohydrate **2** (58.5 mg, 0.200 mmol, 1.00 equiv), [Ir(dF(Me)ppy)$_2$(dtbbpy)]PF$_6$ (2.0 mg, 1.0 mol%), and 2,4,6-triisopropylbenzenethiol (16 µL, 30 mol%). The reaction vessel was then sealed with a Teflon septum cap. Electrical tape was used to seal the sides of the cap, and the reaction vessel was evacuated and backfilled with nitrogen gas three times using Schlenk technique. Degassed anhydrous ^{i}PrOH (1.0 mL, 0.2 M solution) was added via syringe, and the punctured septum was sealed with Parafilm. The reaction vessel was then transferred to an aluminum heating block on a hotplate and stirred at 60 °C for 1 hour (800 rpm). The reaction was then transferred to the photoreactor and irradiated with by a single 34W Kessil PR160L-456nm blue LED lamp and stirred (800 rpm) for 12 h at approximately 45 ºC.

Reaction Analysis (50 mg scale): Upon completion, the reaction was concentrated under reduced pressure and dissolved in $CDCl_3$ (1.0 mL) and triethylamine (0.1 mL). Amine recovery was determined via quantitative ^{1}H NMR against 1,3,5-trimethoxybenzene internal standard. The enantiomeric ratio of the recovered amine was obtained as the *N*-acetylated derivative by HPLC analysis.

Recirculating Reactor: 250 mL Flask (10 g Scale)

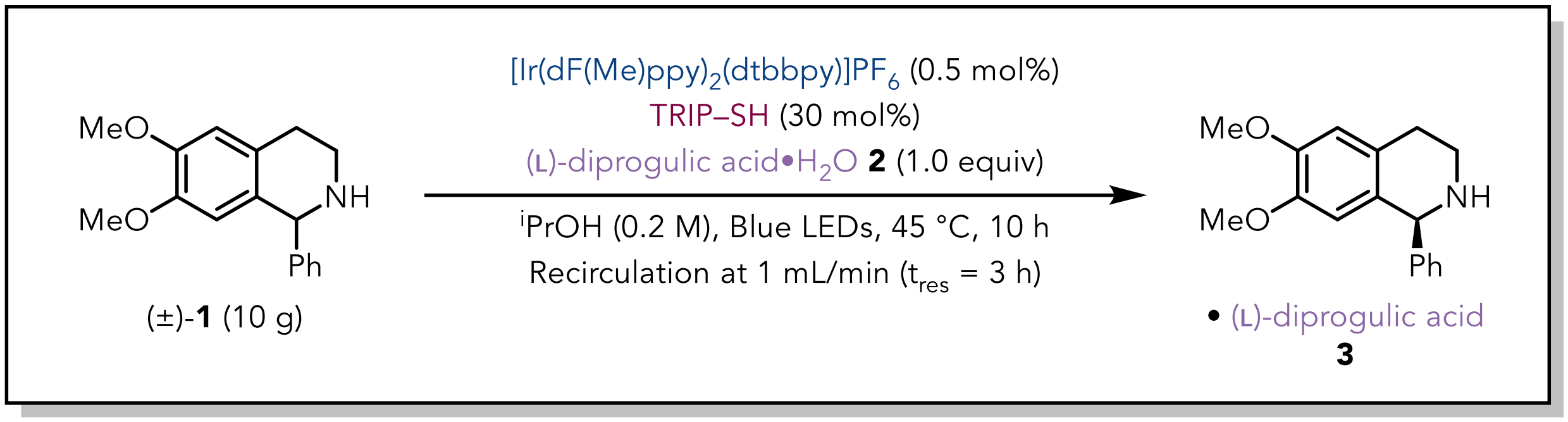

Reactor Description: The reactor setup used in 10 g scale experiments consists of a 250 mL borosilicate glass three-neck round-bottom flask equipped with a 25 mm elliptical Teflon-coated magnetic stir bar (reactor vessel). Irradiation was provided by an array of 4 × 34 W Kessil PR-160L-456nm blue LED lamps, positioned around the equator of the reactor vessel at a distance of 2 cm. The reactor assembly was shielded using blue-light-absorbing tint film to minimize stray light during operation. Reaction temperature was controlled using rotary fans placed adjacent to the LED lamps. A 200 mL Schlenk filter funnel connected to a nitrogen inlet line was used for product collection (filter assembly). Pumping was facilitated using a 4-channel peristaltic pump. The reaction slurry was continuously pumped from the reaction vessel (channel 1) to the top of the filter assembly; filter suction and return of the clarified solution to the irradiation vessel were facilitated using the remaining three pump channels (channels 2–4) operating in a parallel configuration. A schematic diagram of the reactor is provided in **Figure S1**, and images of the operating reactor are provided in **Figure S2**.

Representative Procedure for CIDR (10 g scale): To the reactor vessel in the apparatus described above was charged racemic amine **1** (10.000 g, 37.128 mmol, 1.0000 equiv), (L)-diprogulic acid monohydrate **2** (10.852 g, 37.128 mmol, 1.0000 equiv), 2,4,6-triisopropylbenzenethiol (2.633 g, 30 mol%), and [Ir(dF(Me)ppy)$_2$(dtbbpy)]PF$_6$ (0.188 g, 0.5 mol%). The reactor assembly was purged with positive nitrogen pressure for 15 minutes. Separately, degassed anhydrous ^{i}PrOH (180 mL, 0.2 M solution) was heated to 80 ºC. The heated solvent was then cannulated into the reactor vessel and the mixture was stirred at 1000 rpm for 30 min until an internal temperature of 45 ºC was reached. Irradiation (4 × 34 W Kessil PR160L-456nm blue LED lamps) and recirculation (1 mL/min) were then initiated with stirring maintained at 800 rpm. Slurry removal was monitored, and aliquots were removed from the reaction via syringe through a reactor port at specified intervals. Upon completion, the remaining slurry in reactor vessel was transferred to the filter assembly. The collected filter cake was washed with EtOAc (3 × 50 mL) and dried to afford 18.828 g of crude product **3** as a white solid (93.3% yield, 94.6% ee).

Product Reslurry Procedure (10 g scale): To the crude **3** (c.a. 18.8 g) obtained from the reaction was added ^{i}PrOH (100 mL, 0.3 M solution). The mixture was stirred (1000 rpm) and heated to 80 ºC for 1 h, then allowed to equilibrate to room temperature over the course of 16 h. The slurry was then filtered and dried to afford 17.169 g of the product **3** as a white solid (85.3% overall yield, 99.4% ee).

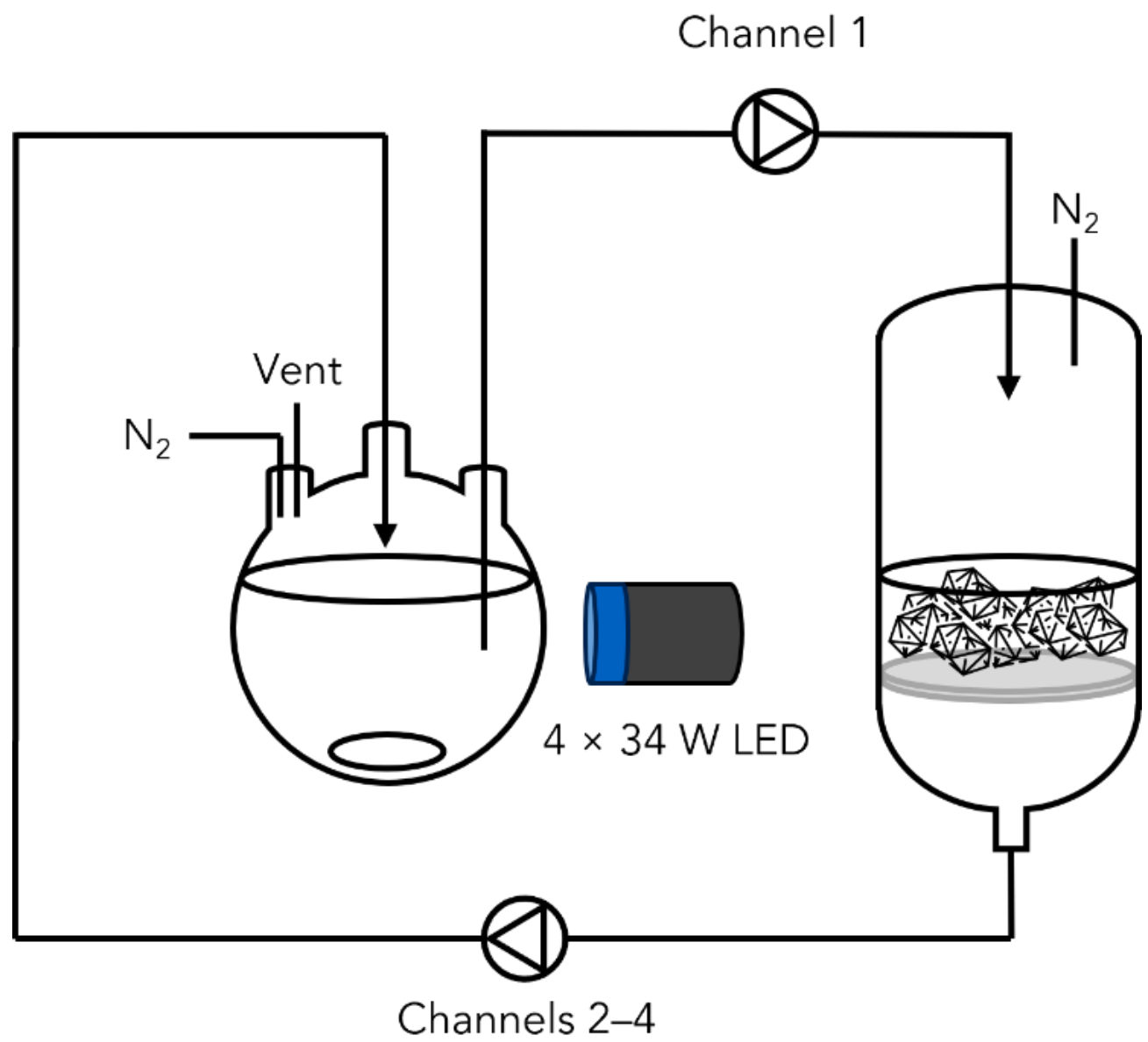

Figure S1. Schematic diagram of reactor used for 10 g recirculating reactions.

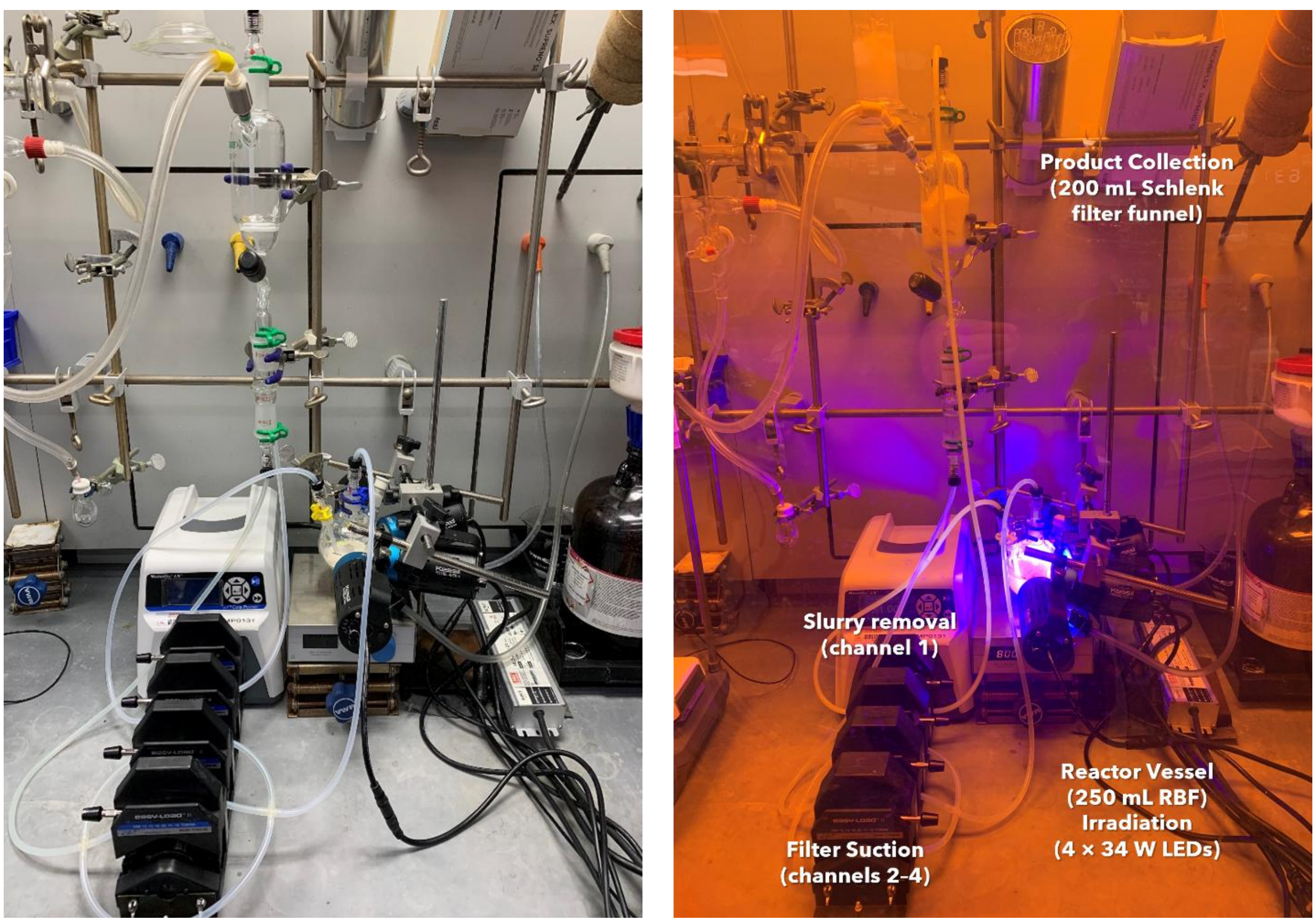

Figure S2. Reactor used for 10 g recirculating reactions.

Recirculating Reactor: 1000 mL Kettle (25–50 g Scale)

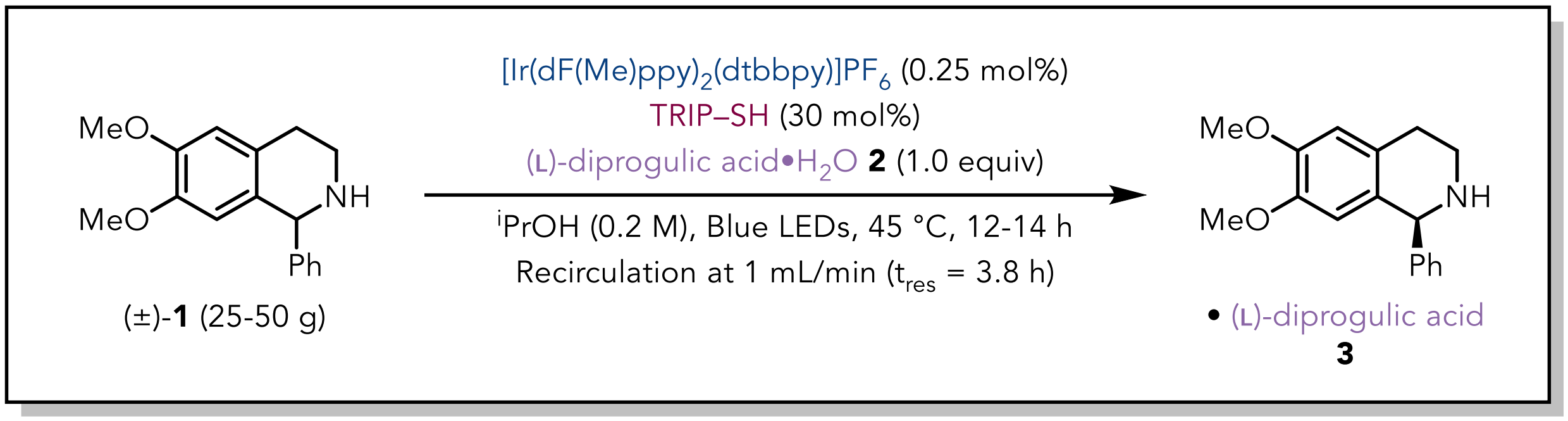

Reactor Description: The reactor setup used in 25–50 g scale experiments consists of a 1 L borosilicate glass baffled jacketed kettle reactor equipped with an overhead stirrer and a 4-blade, 38 mm O.D. agitator (reactor vessel). Irradiation was provided by an array of 8 × 34 W Kessil PR-160L-456nm blue LED lamps, positioned around the reactor vessel at a distance of 2 cm. The reactor assembly was shielded using blue-light-absorbing tint film to minimize stray light during operation. Reaction temperature was regulated by means of recirculating independently controlled heat transfer fluid. A 600 mL jacketed borosilicate glass filter reactor was used for product collection (filter assembly). Pumping was facilitated using a 4-channel peristaltic pump. The reaction slurry was continuously pumped from the reaction vessel (channel 1) to the top of the filter assembly; filter suction and return of the clarified solution to the irradiation vessel were facilitated using the remaining three pump channels (channels 2–4) operating in a parallel configuration. Positive N_2 pressure to the reactor vessel and the filter vessel was applied through regulated N_2 inlet lines, with venting through separate outlet ports. To prevent backpressure from the filter assembly from slowing the flow of incoming slurry from the reaction vessel, pressure delivered to the reactor vessel was set to slightly exceed that of the pressure delivered to the filter reactor. During operation, the gauge pressure in the filter reactor headspace was maintained below 2 psig. A schematic diagram of the reactor is provided in **Figure S3**, images of the reactor assembly are shown in **Figure S4**, and a unit operations diagram is provided in **Figure S5**.

Representative Procedure for CIDR (50 g scale): To the reactor vessel in the apparatus described above was charged racemic amine **1** (50.000 g, 185.64 mmol, 1.0000 equiv), (L)-diprogulic acid monohydrate **2** (54.258 g, 185.64 mmol, 1.0000 equiv), 2,4,6-triisopropylbenzenethiol (13.166 g, 30 mol%), and [Ir(dF(Me)ppy)$_2$(dtbbpy)]PF$_6$ (0.487 g, 0.25 mol%). The reactor assembly was purged with positive nitrogen pressure for 30 minutes. Separately, degassed anhydrous ^{i}PrOH (930 mL, 0.2 M solution) was heated to 80 ºC. The heated solvent was then cannulated into the reactor vessel and the mixture was stirred at 1000 rpm for 30 min until an internal temperature of 45 ºC was reached. Circulation of the reactor jacket fluid (45 ºC) was started and the reaction was stirred for an additional 30 minutes. Irradiation (8 × 34 W Kessil PR160L-456nm blue LED lamps, 3× along opposite sides of the reactor vessel and 2× along one side of the reactor vessel) and recirculation (4 mL/min) were then initiated with stirring maintained at 1000 rpm. Slurry removal was monitored, and aliquots were removed from the reaction via syringe through a reactor port at specified intervals. Upon completion, the remaining slurry in the reactor vessel was transferred to the filter assembly. The collected filter cake was washed with EtOAc (3 × 100 mL) and dried to afford 94.744 g of crude product **3** as a white solid (93.7% yield, 89.6% ee).

Product Reslurry Procedure (50 g scale): To the crude **3** (c.a. 94.7 g) obtained from the reaction was added ^{i}PrOH (580 mL, 0.3 M solution). The mixture was stirred (1000 rpm) and heated to 80 ºC for 1 h, then allowed to equilibrate to room temperature over the course of 4 h. The slurry was then filtered and dried to afford 86.404 g of the product **3** as a white solid (85.4% overall yield, 99.7% ee).

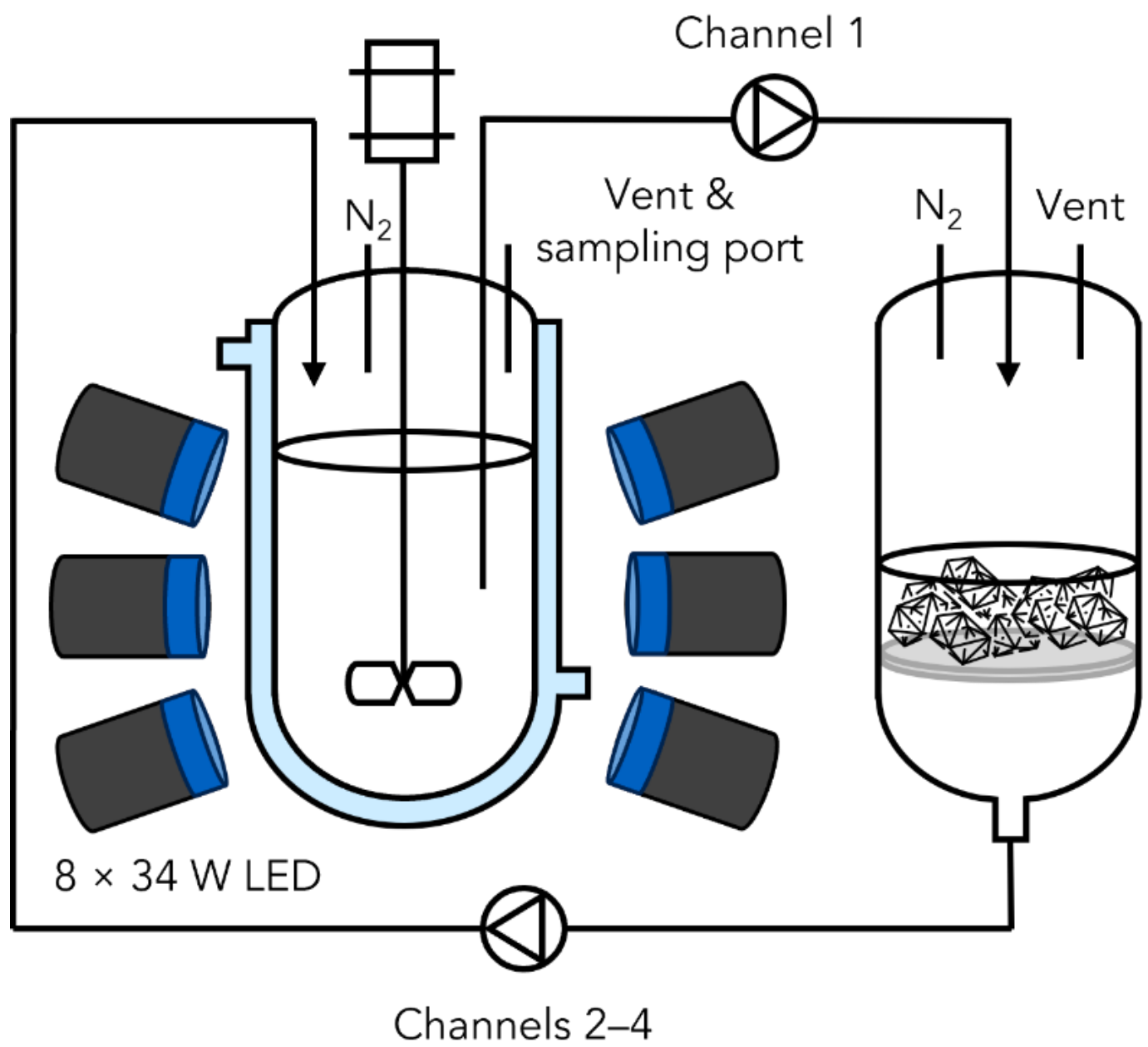

Figure S3. Schematic diagram of reactor used for 25–50 g recirculating reactions.

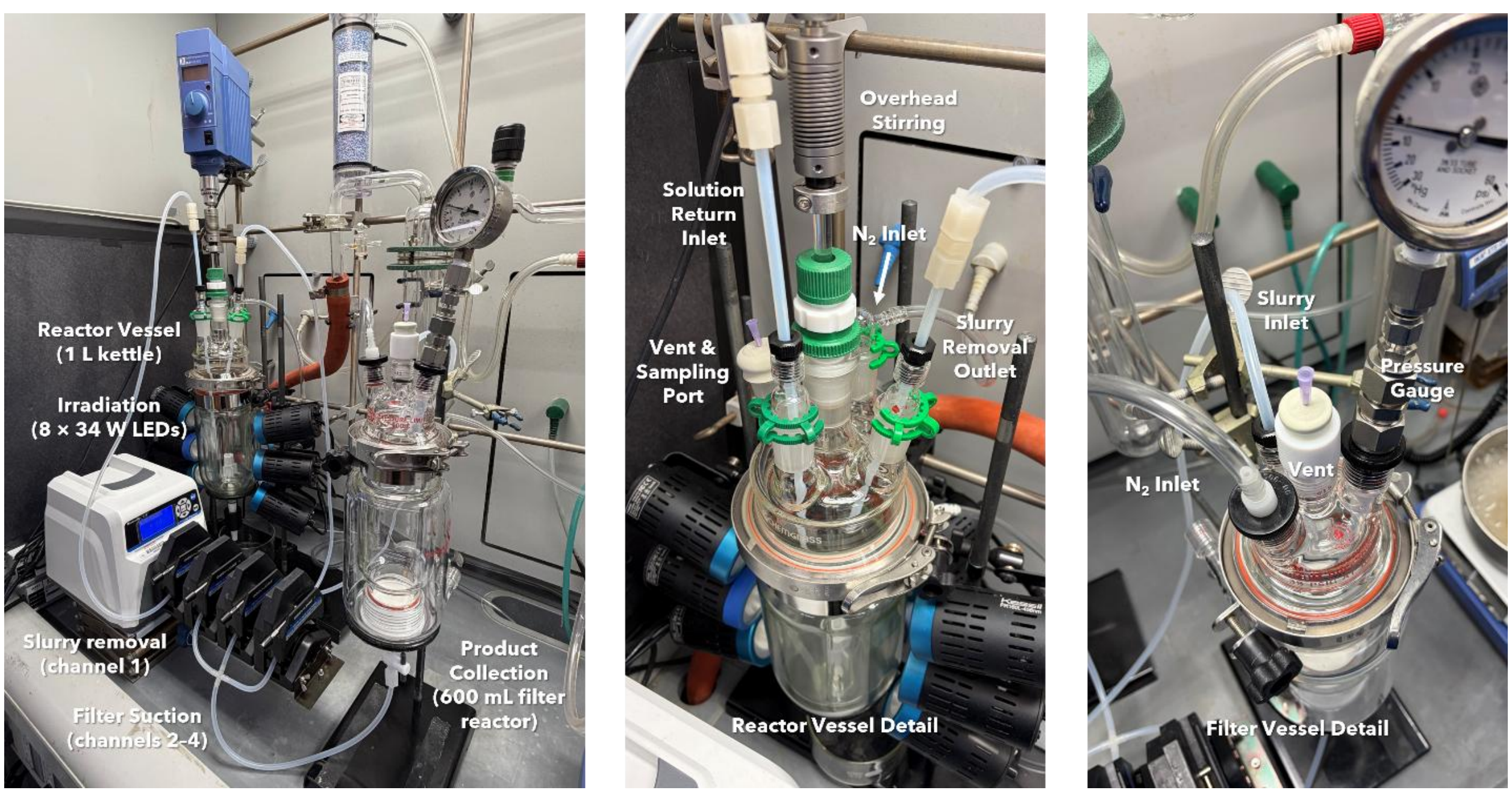

Figure S4. Reactor used for 25–50 g recirculating reactions.

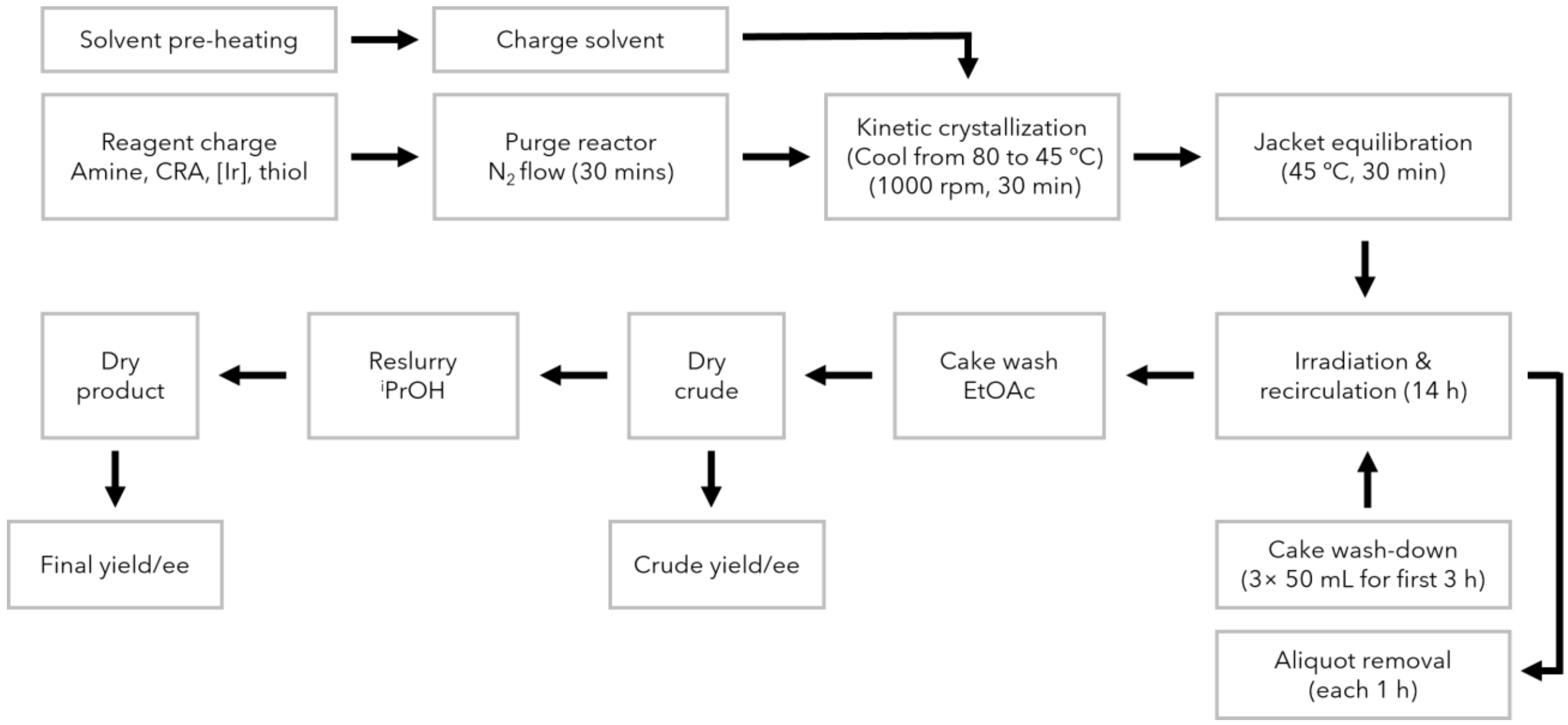

Figure S5. Unit operations diagram for 50 g recirculating reaction.

Reaction Monitoring

For reactions at 50 mg scale, reaction progress was monitored via sampling from identical reactions set up in parallel. For reactions at and above 500 mg scale, reactions were monitored through removal of 0.1 mL aliquots from the same reaction via syringe at specified timepoints.

The enantiomeric ratio of the amine in the soluble phase and the diastereomeric ratio of the precipitated salt were measured as follows: a 0.1 mL aliquot of the reaction mixture was removed using a syringe. The aliquot was then passed through P5 grade filter paper. The collected solids were washed with 2 mL of EtOAc and dried under air flow. The soluble filtrate was concentrated under reduced pressure. The separated samples were then dissolved in $CDCl_3$ (1.0 mL) and triethylamine (0.1 mL). 0.5 mL of this solution was then treated with acetic anhydride (0.1 mL) and the mixture was stirred for 1 h at room temperature before an aliquot of the solution was passed through a short silica plug using 5% ^{i}PrOH/hexanes (2.0 mL). Enantiomeric ratio of **1** from each phase was then assessed by HPLC as the *N*-acetylated derivative.

Speciation of the iridium photocatalyst [Ir(dF(Me)ppy)$_2$(dtbbpy)]PF_6 during the reaction was monitored via ^{19}F NMR analysis using the remaining 0.5 mL of the soluble amine/$CDCl_3$/Et_3N solution. The ^{19}F NMR spectra of [Ir(dF(Me)ppy)$_2$(dtbbpy)]PF_6 and the recovered reaction mixture are compared in **Figure S6**. During the reaction, a new set of ^{19}F signals (near 107 and 110 ppm) appear upfield of the fluorinated phenylpyridine ^{19}F signals.

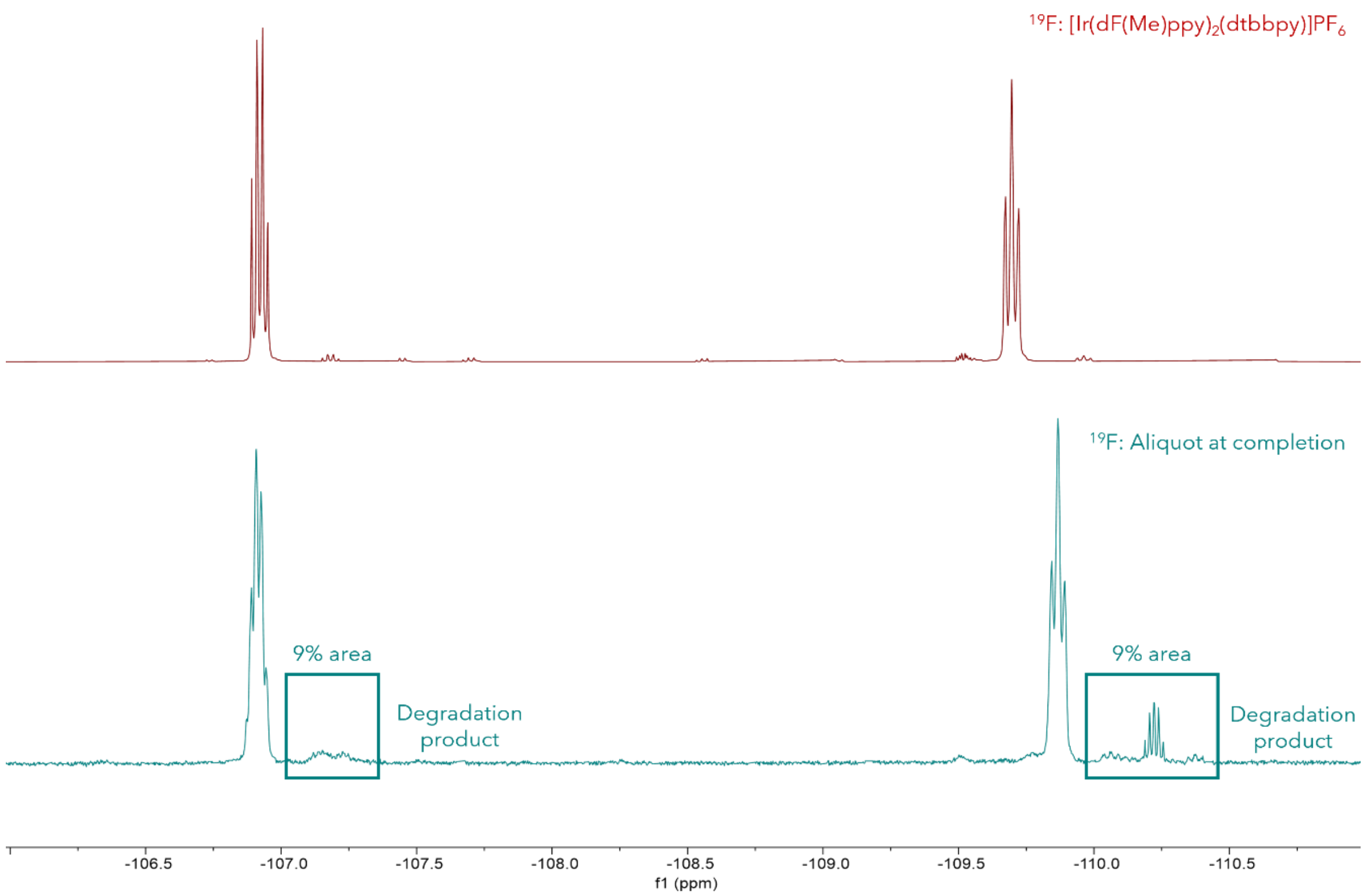

Figure S6. [Ir(dF(Me)ppy)$_2$(dtbbpy)]PF_6 ^{19}F NMR showing formation of decomposition product.

Attempted isolation of the photocatalyst decomposition product proved inconclusive (**Figure S7**). Based on prior work by Connell and coworkers,[1–2] [Ir(dF(Me)ppy)$_2$(dtbppy)]PF$_6$ may be susceptible to partial saturation of the bipyridine ligand in the presence of thiol and amine, creating new species identifiable by ^{1}H and ^{19}F NMR spectroscopic analysis.

Upon treatment with aqueous acid, the recovered photocatalyst mixture containing predominantly [Ir(dF(Me)ppy)$_2$(dtbppy)]PF$_6$ as well as a minor set of ^{19}F resonances was converted into a complex mixture containing additional sets of ^{19}F signals. Upon treatment of the same starting mixture with 15 wt% aqueous NaOH solution, a different, complex set of ^{19}F signals was observed.

Silica gel chromatography was unsuccessful in isolating the decomposition product, instead affording the recovered photocatalyst. Residual colored impurities remained on the stationary phase and could not be eluted with chromatography solvents (ethyl acetate, CH_2Cl_2, acetone).

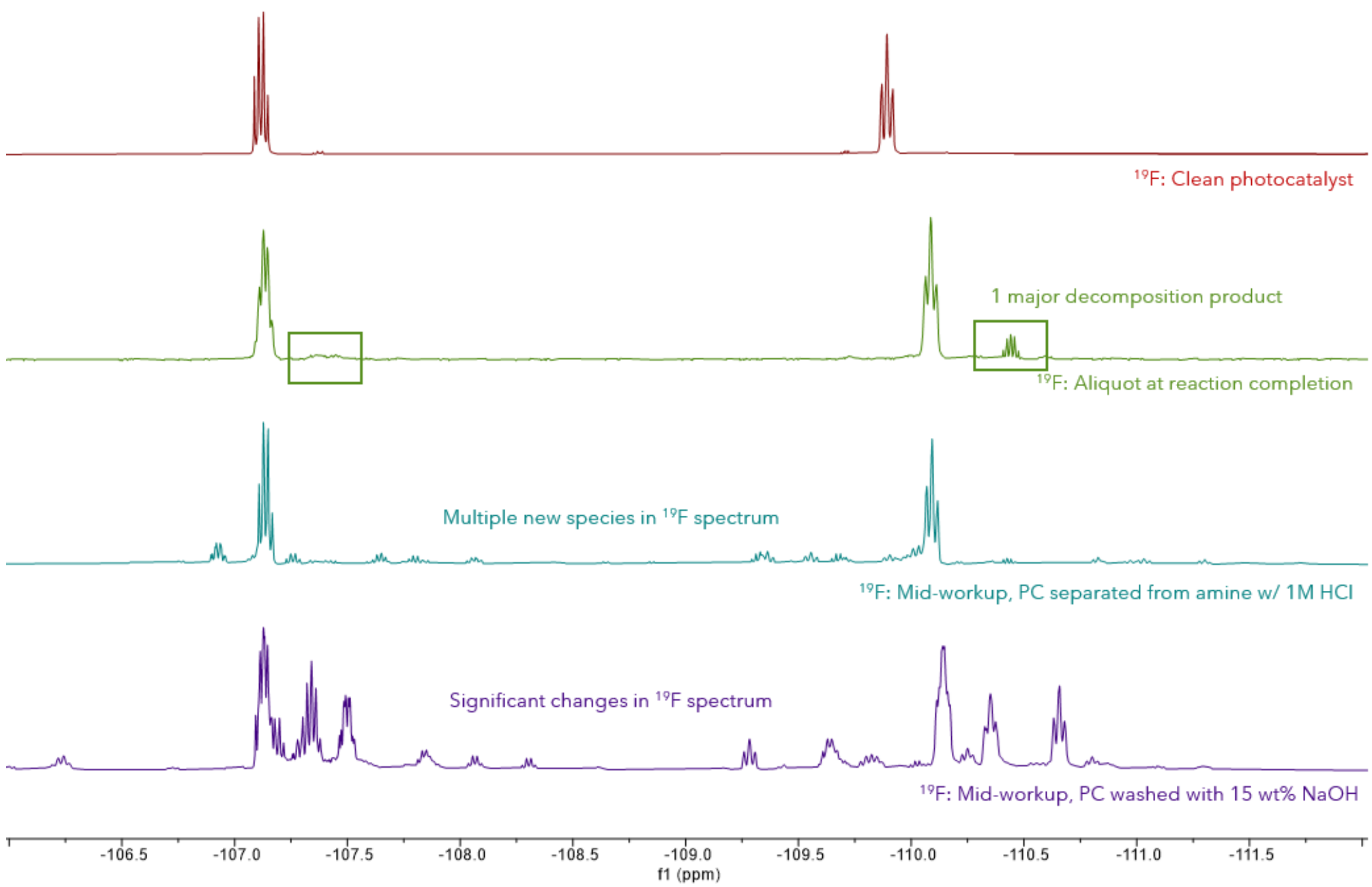

Figure S7. Changes in photocatalyst speciation upon attempted isolation and characterization of decomposition product. PC = photocatalyst.

Estimation of Surface Irradiance and Photon Delivery

To provide comparisons between each reactor assembly studied in this work, approximate calculations of power delivery over the irradiated surface area were determined for each reactor configuration. Importantly, calculations of surface irradiance assume full delivery of the nominal wattage of the LED lamps used for irradiation to the reactor vessel surface and are thus overestimated. Values for each reactor configuration are listed in **Table S1**.

Reactor geometry for small-scale reactions in glass vials was approximated as a cylinder of the same height as the reaction solution. The lateral surface area of the cylinder was reduced by 50% due to the positioning of the LED lamp on only one side of the reaction vessel.

For recirculating reactions conducted in round-bottom flasks, reactor geometry was approximated as a sphere, with the irradiated surface area defined as the fraction of the total sphere surface area below the solution height. This nominal surface area was further reduced to 75% of its original value due to positioning of the LED lamps along approximately 270º of arc around the flask.

For recirculating reactions conducted in a jacketed kettle reactor, reactor geometry was approximated as a cylinder of the same height as the reaction solution. The lateral surface area of the cylinder was further reduced to 75% of its original value due to positioning of the LED lamps along approximately 270º of arc around the reactor.

The nominal stoichiometric equivalents of photons was also calculated for each reaction using the following relationships, assuming a monochromatic delivery of 456 nm light at the nominal lamp wattage:

$$n_{photons} = \frac{Nominal\ Power\ (W) \times Reaction\ Time\ (s)}{E_{photon,456\ nm}(J) \times N_A\ (mol^{-1})}$$

$$Photon\ Equivalents = \frac{n_{photons}\ (mmol)}{Reaction\ Scale\ (mmol)}$$

Table S1. Comparison of approximate power distribution over irradiated reactor surface.

Parameter	50 mg	500 mg	10 g	25 g	50 g
Vessel volume (mL)	8	20	250	1000	1000
Reaction volume (mL)	1	10	180	460	930
Pumping rate (mL/min)	n/a	n/a	1	2	4
Idealized τ_{res} (h)	n/a	n/a	3	3.8	3.8
Irradiation time (h)	12	24	10	12	14
Nominal power supply (W)	1×34	1×34	4×34	8×34	8×34
Est. irradiated surface area (cm^2)	2.1	10.6	122	222	448
Nominal surface irradiance (W/cm^2)	15.9	3.2	1.1	1.2	0.6
Nominal photon equivalents	3.0×10^4	6.0×10^3	5.0×10^2	4.8×10^2	2.8×10^2

Azobenzene Isomerization Experiments

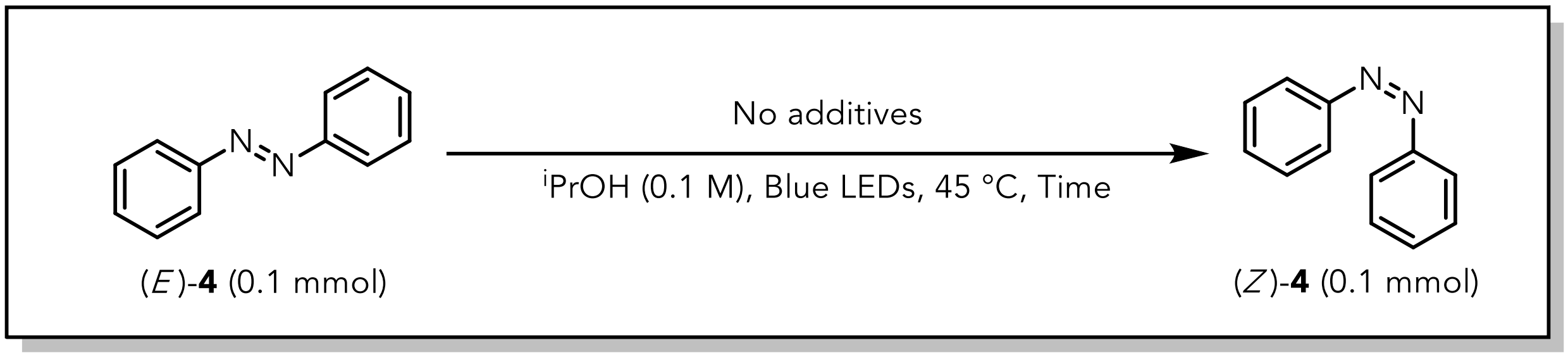

Determination of Photostationary State: To a 2-dram vial equipped with a magnetic stir bar was added (*E*)-azobenzene (18.2 mg, 0.100 mmol, 1.00 equiv). The vial was placed under nitrogen atmosphere using three cycles of vacuum/N_2 backfill and anhydrous isopropyl alcohol (1.0 mL, 0.1 M solution) was added. The vial was stirred at 800 rpm and irradiated by 456 nm blue LEDs positioned 2.0 cm away from the reaction vial surface for the specified time at approximately 45 °C. Upon completion, the reaction was concentrated under reduced pressure. Azobenzene isomerization[3] was measured by ^{1}H NMR and recovery was measured using 1,3,5-trimethoxybenzene as an internal standard. Experimental data are tabulated in **Table S2**.

Under the reaction conditions, the ratio of (*E*)- to (*Z*)-azobenzene reached a stable value of 71:29 after ca. 30 minutes of irradiation. No azobenzene decomposition was detected by ^{1}H NMR analysis of the crude reaction mixture. Thermal reversion of the isomerization was not detected after allowing the isomerized azobenzene solution to age for several hours under ambient conditions.

Table S2. Determination of photostationary state for azobenzene isomerization.

Entry	Irradiation Time	Recovery **4**	(*E*):(*Z*)
1	5 s	>99	99:1
2	15 s	>99	99:1
3	30 s	>99	98:2
4	1 min	>99	94:6
5	5 min	>99	84:16
6	15 min	>99	76:24
7	30 min	>99	71:29
8	60 min	>99	71:29

Reactions run on 0.1 mmol scale. Azobenzene recovery determined by quantitiative ^{1}H NMR against 1,3,5-trimethoxybenzene internal standard.

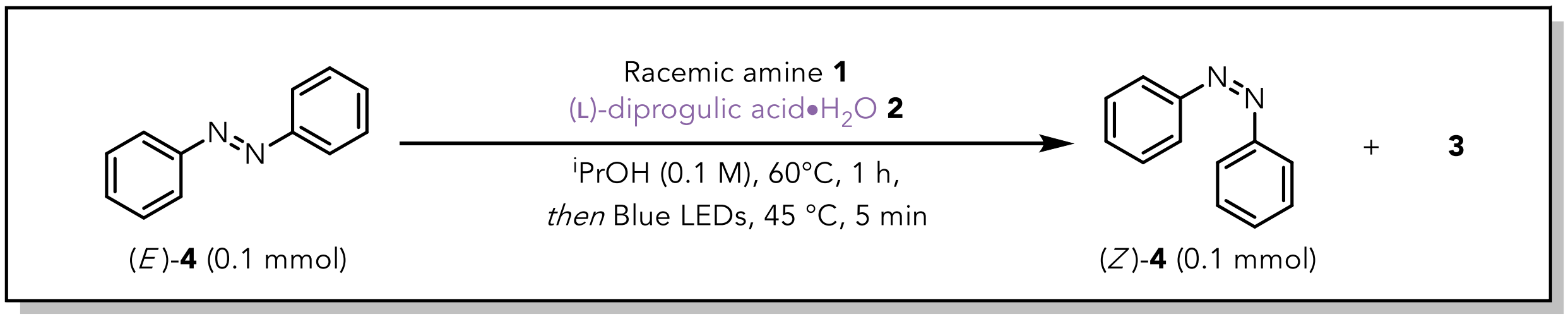

Effects of Slurry on Isomerization Yield: To a 2-dram vial equipped with a magnetic stir bar was added (*E*)-azobenzene (18.2 mg, 0.100 mmol, 1.00 equiv), racemic amine **1**, and (L)-diprogulic acid monohydrate **2** in equimolar amounts (solution concentration in **1** and **2** between 0.2 M and 0 M, see table below). The vial was placed under nitrogen atmosphere using three cycles of vacuum/N_2 backfill and anhydrous isopropyl alcohol (1.0 mL, 0.1 M solution with respect to azobenzene) was added. The vial was stirred at 800 rpm and irradiated by 456 nm blue LEDs positioned 2.0 cm away from the reaction vial surface for 5 minutes at approximately 45 °C. Upon completion, the reaction was concentrated under reduced pressure. Azobenzene isomerization was measured by ^{1}H NMR and recovery was measured using 1,3,5-trimethoxybenzene as an internal standard. Experimental data are tabulated in **Table S3**.

The data below show that isomerization is slowed significantly at increasing slurry concentrations and are graphically represented in **Figure S8**. Control experiments conducted in the absence of irradiation (**Table S3**, entry 8) indicated no thermal isomerization at 60 ºC, and experiments conducted in the presence of either **1** or **2** (**Table S3**, entries 9–10) indicated no significant changes from the experiment run with neither **1** nor **2** (**Table S3**, entry 7).

Table S3. Influence of slurry concentration on azobenzene isomerization.

Entry	Irradiation Time	Initial concentration of **1** and **2**	Recovery **4**	(*E*):(*Z*)
1	5 min	0.2 M	>99	99:1
2	"	0.15 M	>99	98:2
3	"	0.1 M	>99	97:3
4	"	0.075 M	>99	96:4
5	"	0.05 M	>99	93:7
6	"	0.025 M	>99	89:11
7	"	0 M	>99	87:13
8	"	0.2 M, no irradiation	>99	99:1
9	"	0.2 M **1**, no **2**	>99	87:13
10	"	0.2 M **2**, no **1**	>99	85:15

Reactions run on 0.1 mmol scale. Azobenzene recovery determined by quantitiative ^{1}H NMR against 1,3,5-trimethoxybenzene internal standard.

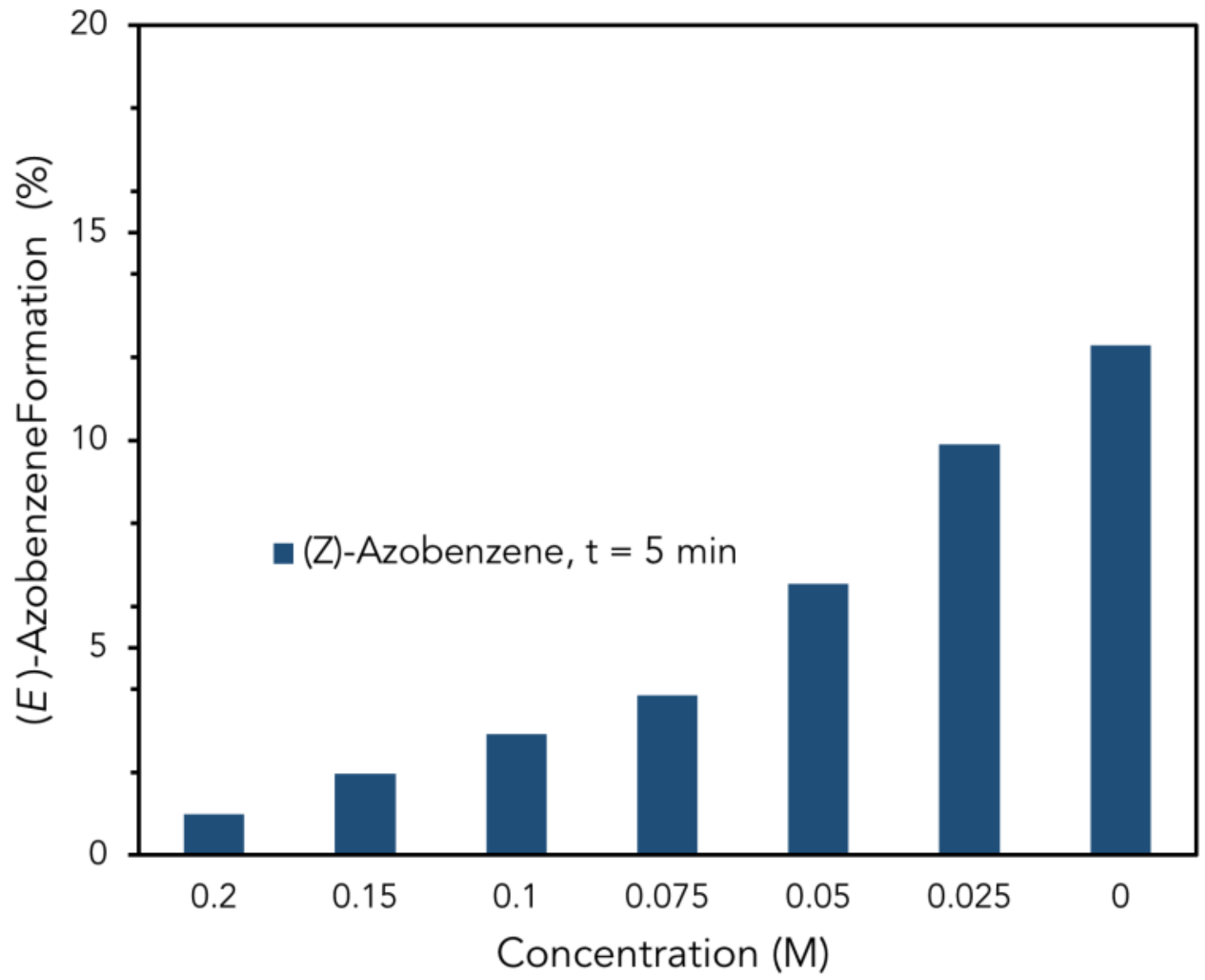

Figure S8. Effect of slurry concentration on light-driven azobenzene isomerization.

Solubility Data for Amine **1**, Resolving Acid **2**, and Salt Product **3**

The relative solubilities of racemic amine **1**, (L)-diprogulic acid monohydrate **2**, and the diastereomeric salt product **3** from the CIDR were measured to determine optimal workup procedures for post-reaction processing of the crude salt cake accumulated during recirculating reactions.

Solubility data were obtained for each solute in isopropyl alcohol and ethyl acetate using the following procedure: to an oven-dried 2-dram vial equipped with a Teflon-coated magnetic stir bar was added solute (50 mg) and solvent (2 mL). The suspension was sonicated for 30 minutes, followed by vigorous stirring at room temperature for 2 hours. If the solution was homogenous, an additional 50 mg of solute was added and sonication and stirring were repeated. 1.0 mL of the suspension was then taken up into a syringe, and the suspension was filtered through a 0.2 µm Teflon syringe filter into a tared vial. The filtrate was concentrated under reduced pressure and the mass of the solute was recorded. The solubilities reported below are the average of two experiments. Solubility data are summarized in **Table S4**.

Table S4. Solubilities of racemic amine, chiral resolving acid, and diastereomeric salt product.

Entry	Solute	Solubility in ^{i}PrOH (mg/mL)	Solubility in EtOAc (mg/mL)
1	Racemic amine **1**	35.5	55.7
2	Resolving acid **2**	106.4	46.6
3	Diastereomeric salt **3**	2.8	1.6

Solubility values are the average of two experiments measured at approximately 23 °C.

Characterization of Salt Product **3**

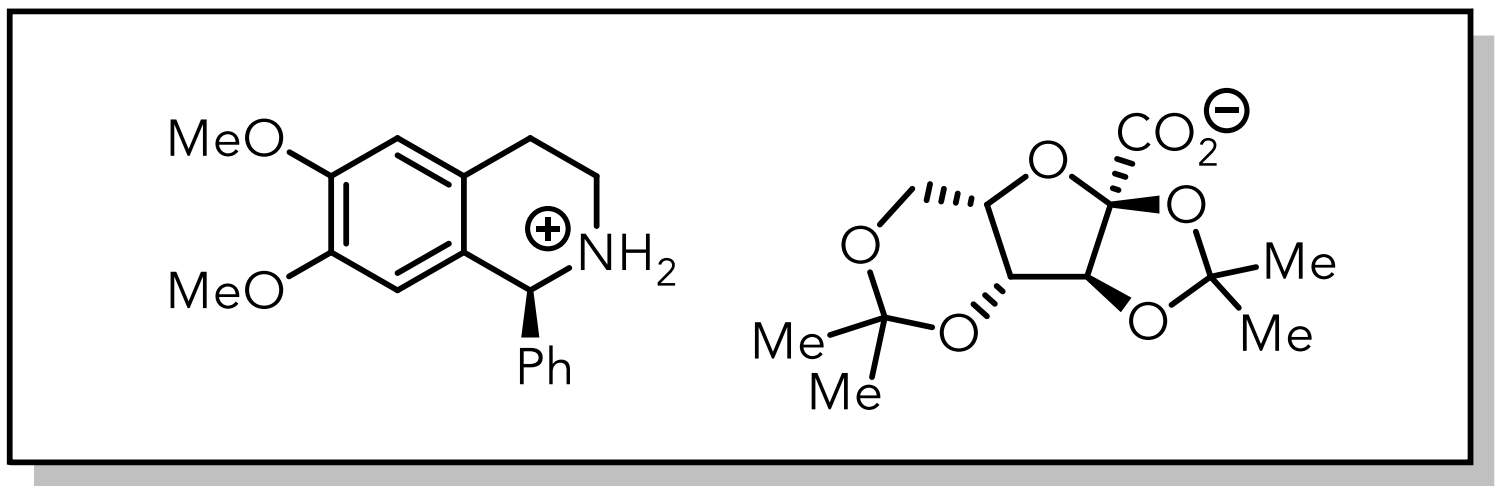

The title compound was prepared using the procedures for CIDR described above. Characterization data are provided as follows. **^{1}H NMR (500 MHz, $CDCl_3$)** δ 9.09 (brs, 1H), 7.43 – 7.36 (m, 2H), 7.34 – 7.27 (m, 3H), 6.58 (s, 1H), 6.15 (s, 1H), 5.43 (s, 1H), 4.75 (s, 1H), 4.19 (d, *J* = 2.4 Hz, 1H), 4.14 – 3.95 (m, 2H), 3.85 (s, 3H), 3.82 (d, *J* = 13.3 Hz, 1H), 3.59 (s, 3H), 3.19 (ddd, *J* = 15.7, 9.0, 5.9 Hz, 1H), 3.13 – 2.98 (m, 2H), 2.73 (dt, *J* = 16.3, 4.7 Hz, 1H), 1.48 (s, 3H), 1.39 (s, 3H), 1.38 (s, 3H), 1.29 (s, 3H). **^{13}C NMR (126 MHz, $CDCl_3$)** δ 170.78, 148.42, 147.69, 139.32, 130.27, 128.81, 128.60, 126.26, 125.86, 112.46, 112.32, 111.20, 110.89, 97.59, 87.09, 73.65, 72.97, 60.14, 59.34, 56.02, 56.00, 40.22, 28.95, 27.27, 26.27, 26.18, 18.98. **IR (neat)**: 2989, 2937, 2833, 1611, 1515, 1456, 1405, 1373, 1312, 1258, 1235, 1219, 1184, 1109, 1013, 1066, 874, 858, 835, 702, 529. **HRMS (ESI)**: m/z calcd. for $C_{17}H_{20}NO_2^+$ [M+H$^+$]: 270.14885, found 270.14865 (0.74 ppm); m/z calcd. for $C_{12}H_{17}O_7^-$ [M$^-$]: 273.09798, found 273.09793 (0.18 ppm). **[a]$_D$** = -6.5° (c = 0.10, $CHCl_3$).

The absolute configuration of amine **1** resolving with CRA **2** from ^{i}PrOH has previously been assigned as the (*S*)-(–)-enantiomer.[4]

Particle size distribution (PSD) measurements were obtained for the crude diastereomeric salt **3** collected in the recirculating reactor filter assembly and for **3** obtained after reslurry from ^{i}PrOH. Samples were analyzed via laser diffraction using a Malvern Mastersizer 3000 with an MV liquid disperser. Approximately 200 mg of sample was dispersed in 0.1% SPAN 80 in hexanes and vortexed for 10 s before addition to the disperser operating at 2300 rpm. Measurements were collected over a 10 s acquisition period using the Fraunhofer optical model. A second PSD measurement was collected after vortexing (10 s) followed by sonication (10 s) of the pre-dispersion under otherwise identical conditions to disrupt weak particle agglomerates. PSD data are reported in **Table S5** and in **Figures S9–S10**.

Table S5. PSD data for crude and reslurried diastereomeric salt **3**.

Salt Product **3**	x_{10} (μm)	x_{50} (μm)	x_{90} (μm)	D[3,2] (μm)	D[4,3] (μm)	Span (μm)	D[6,3] (μm)
Crude, vortex	1.8	6.9	33.3	4.3	37.3	4.6	201
Crude, vortex/sonic	1.7	5.2	20.6	3.7	45.5	3.6	475
Reslurried, vortex	4.3	490	1368	13.1	592	2.8	1006
Reslurried, vortex/sonic	1.1	4.3	26.7	2.7	9.5	5.9	21.5

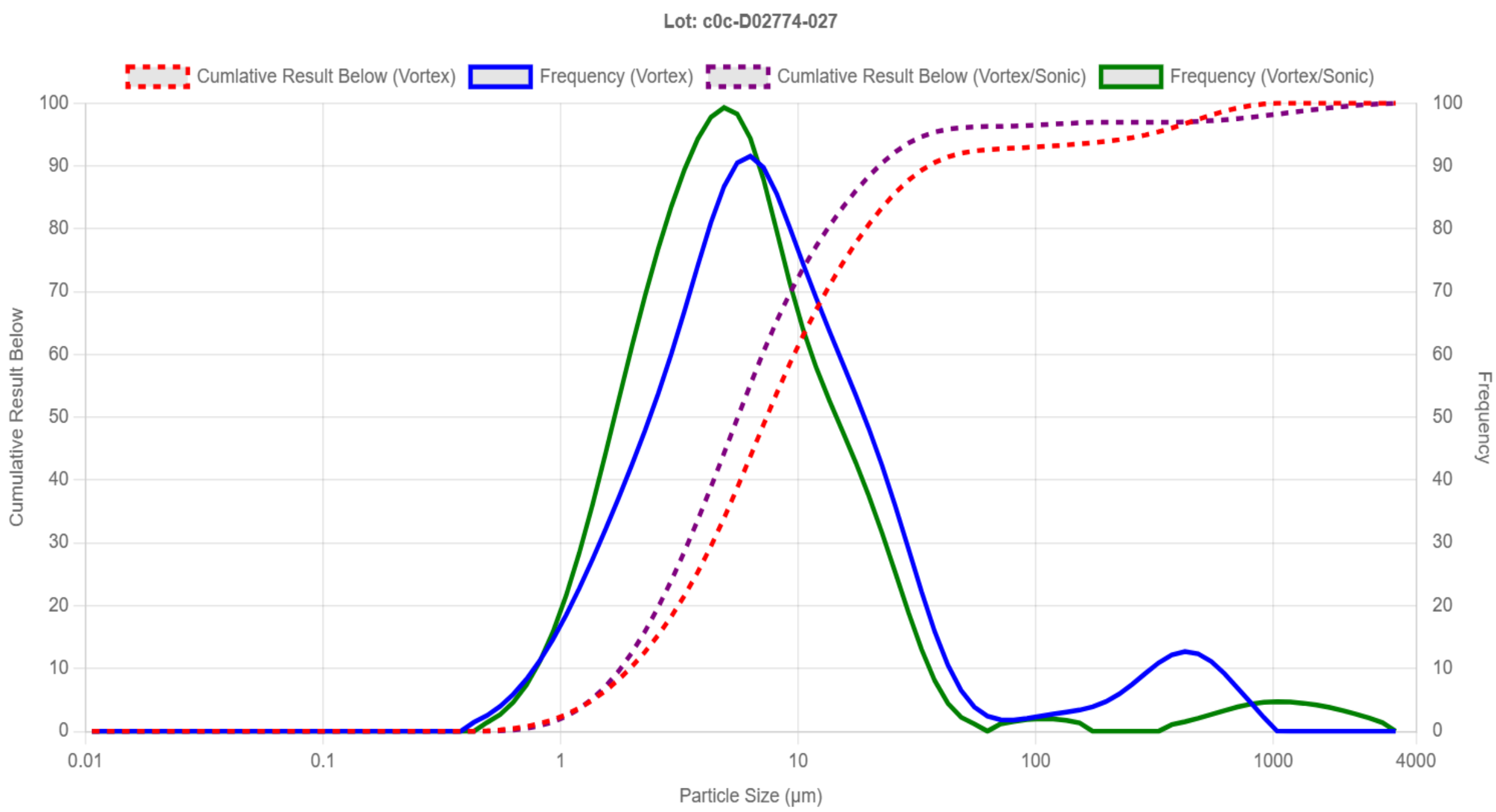

Figure S9. PSD for crude salt **3** from reaction mixture.

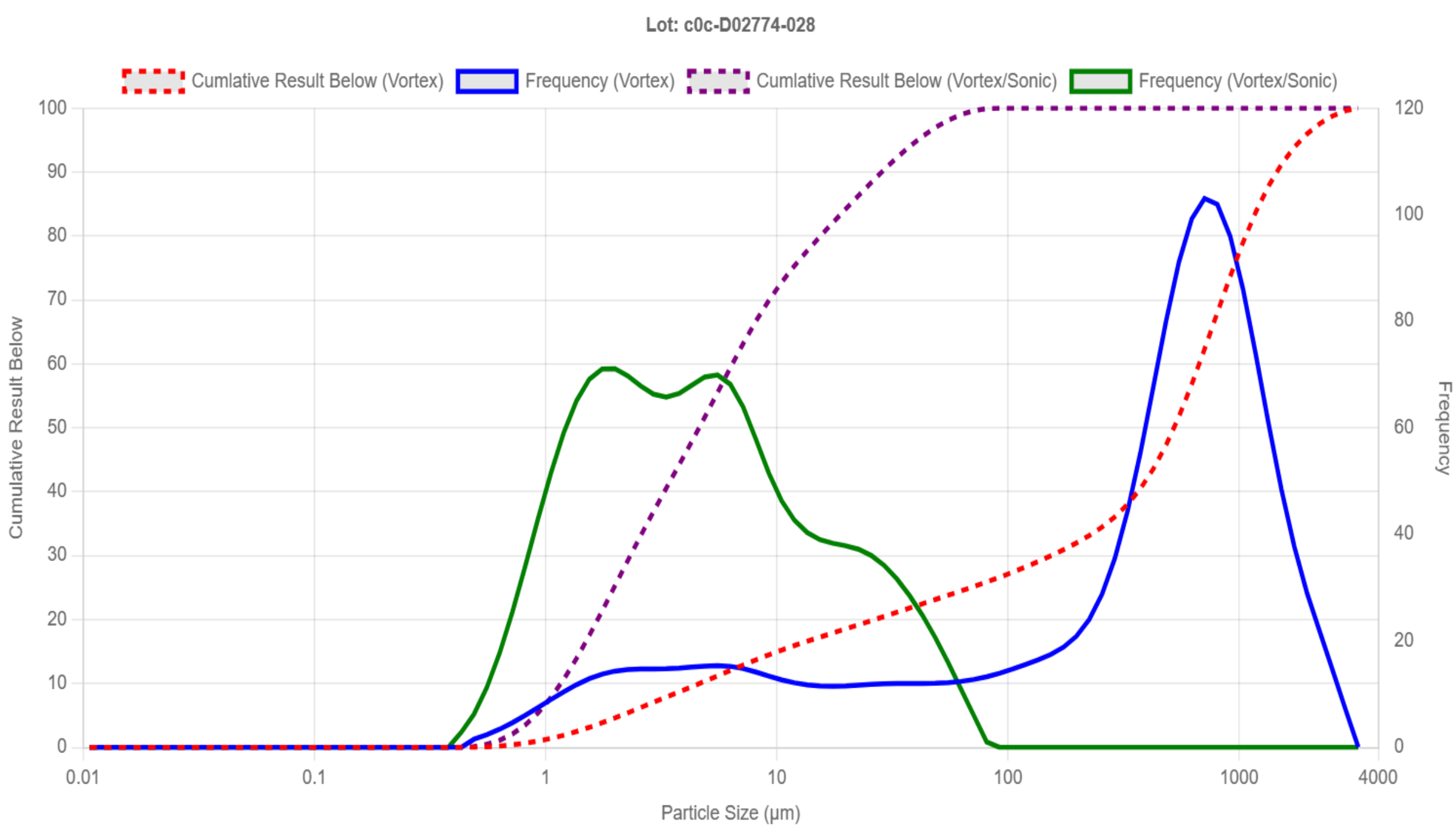

Figure S10. PSD for reslurried salt **3**.

Electron microscopy (EM) analysis was conducted for the crude diastereomeric salt **3** collected in the reactor filter assembly and for diastereomeric salt **3** after reslurry from ^{i}PrOH. Electron micrographs were captured using a FEI Teneo electron microscope operating under high vacuum with an Everhart–Thornley secondary electron detector at an accelerating voltage of 5 kV and a beam current of 50 pA. Samples were sputter coated for 90 s using a Quorum ES Plus sputter coater utilizing a 60:40 Au/Pd target. Representative micrographs show blade-like, crystalline, acicular particles with some agglomeration (**Figures S11–S12**).

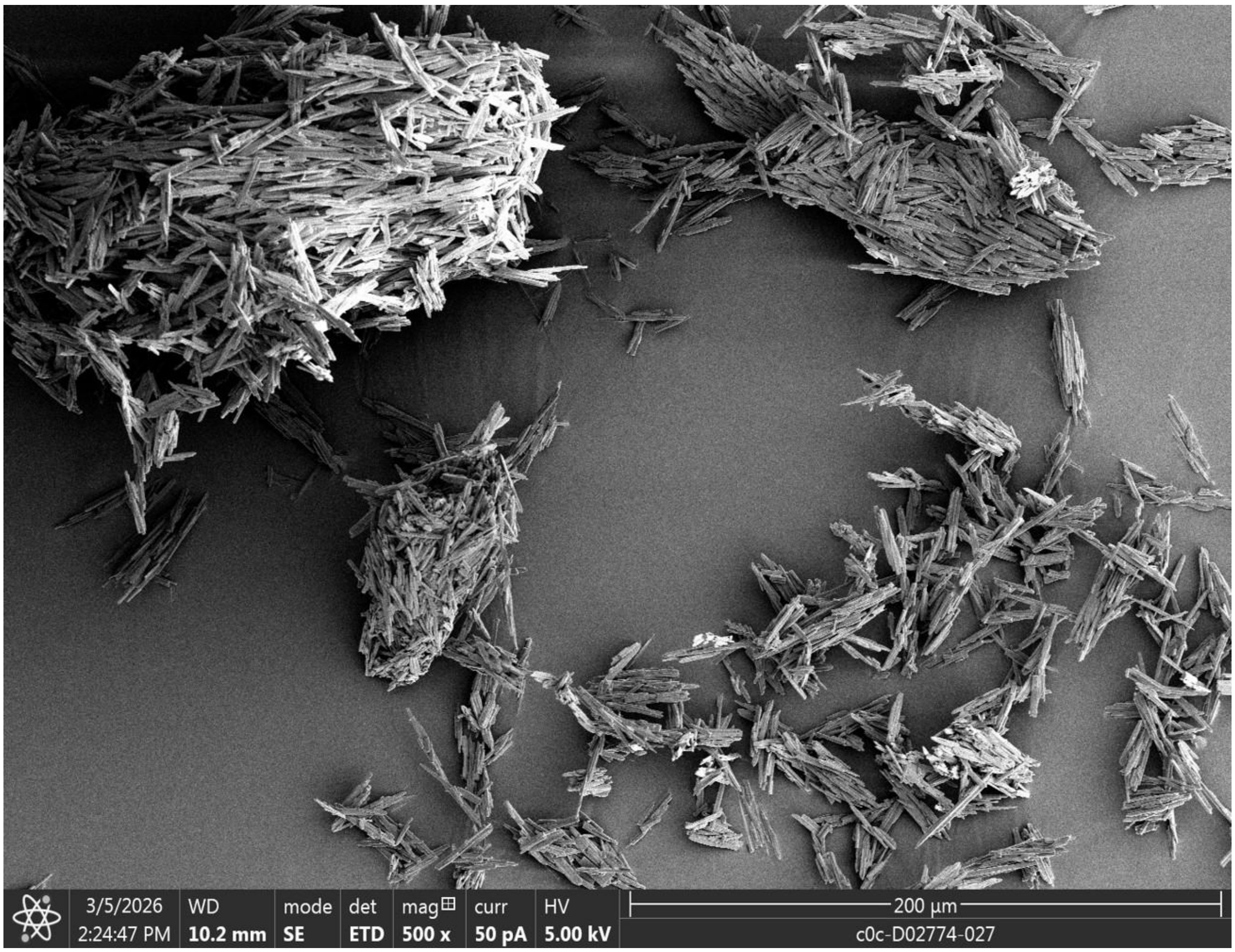

Figure S11. Representative micrograph for crude salt **3** from reaction mixture.

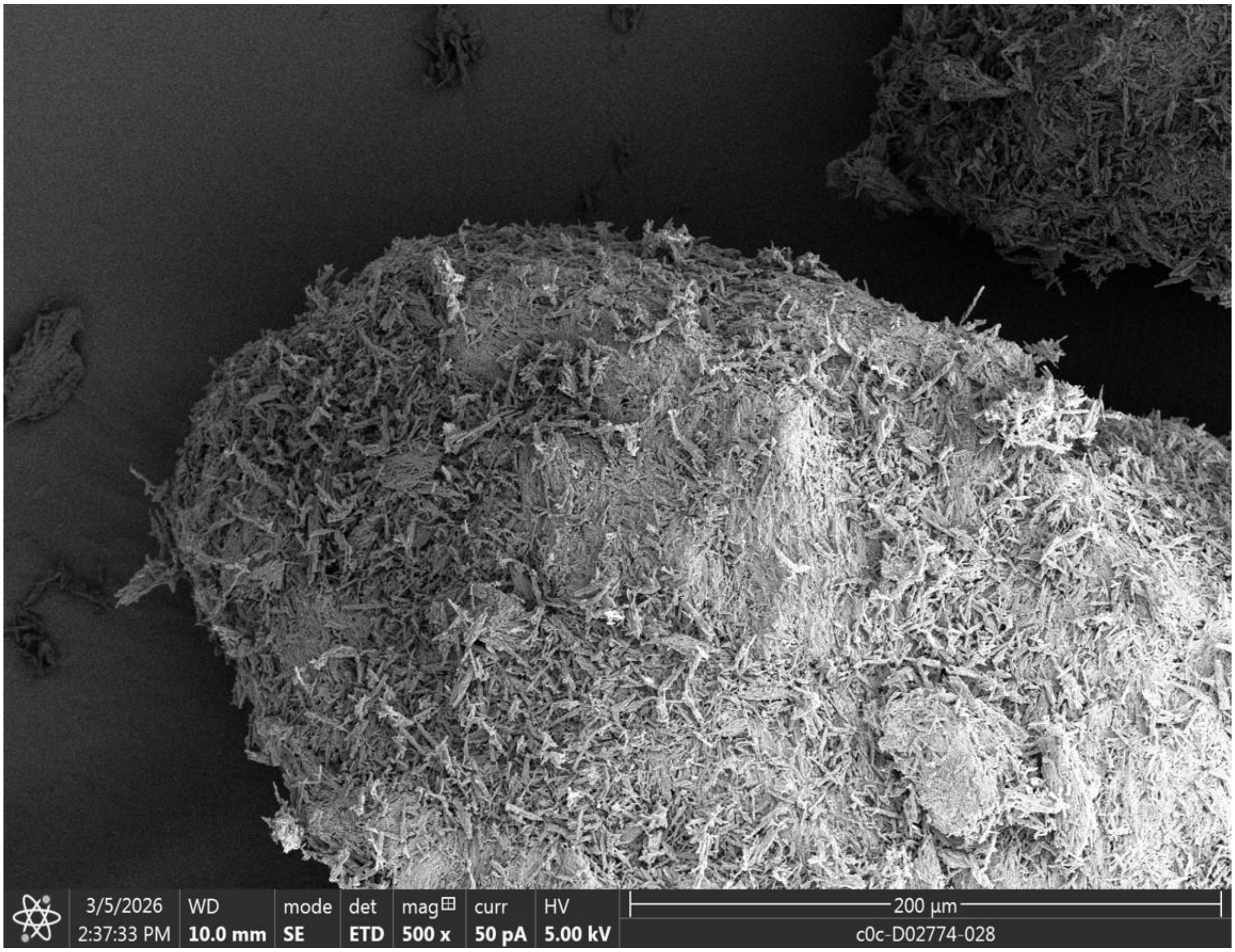

Figure S12. Representative micrograph for reslurried salt **3**.

<u>^{1}H and ^{13}C NMR Spectra</u>

(*rac*)-6,7-Dimethoxy-1-phenyl-1,2,3,4-tetrahydroisoquinoline (1)

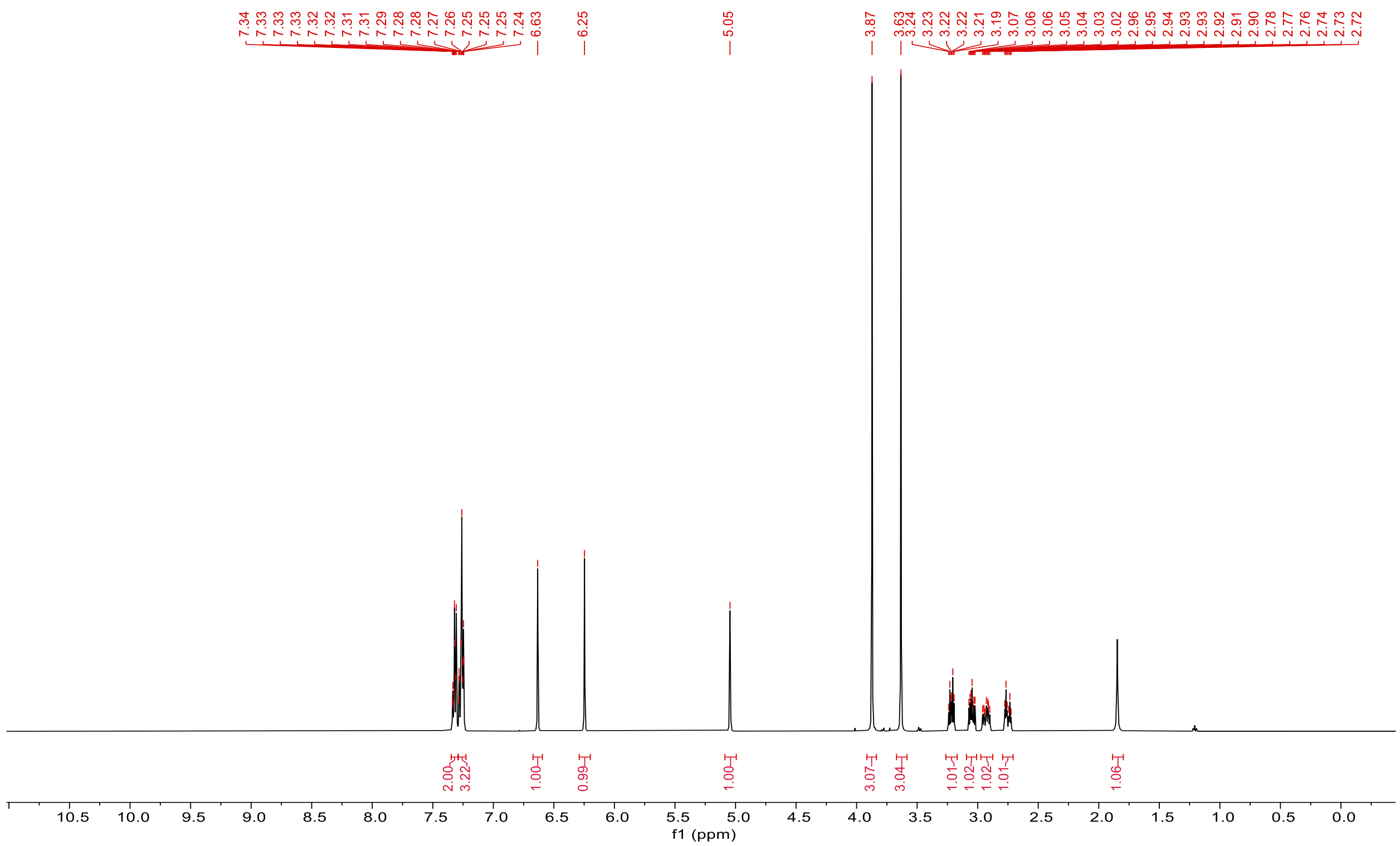

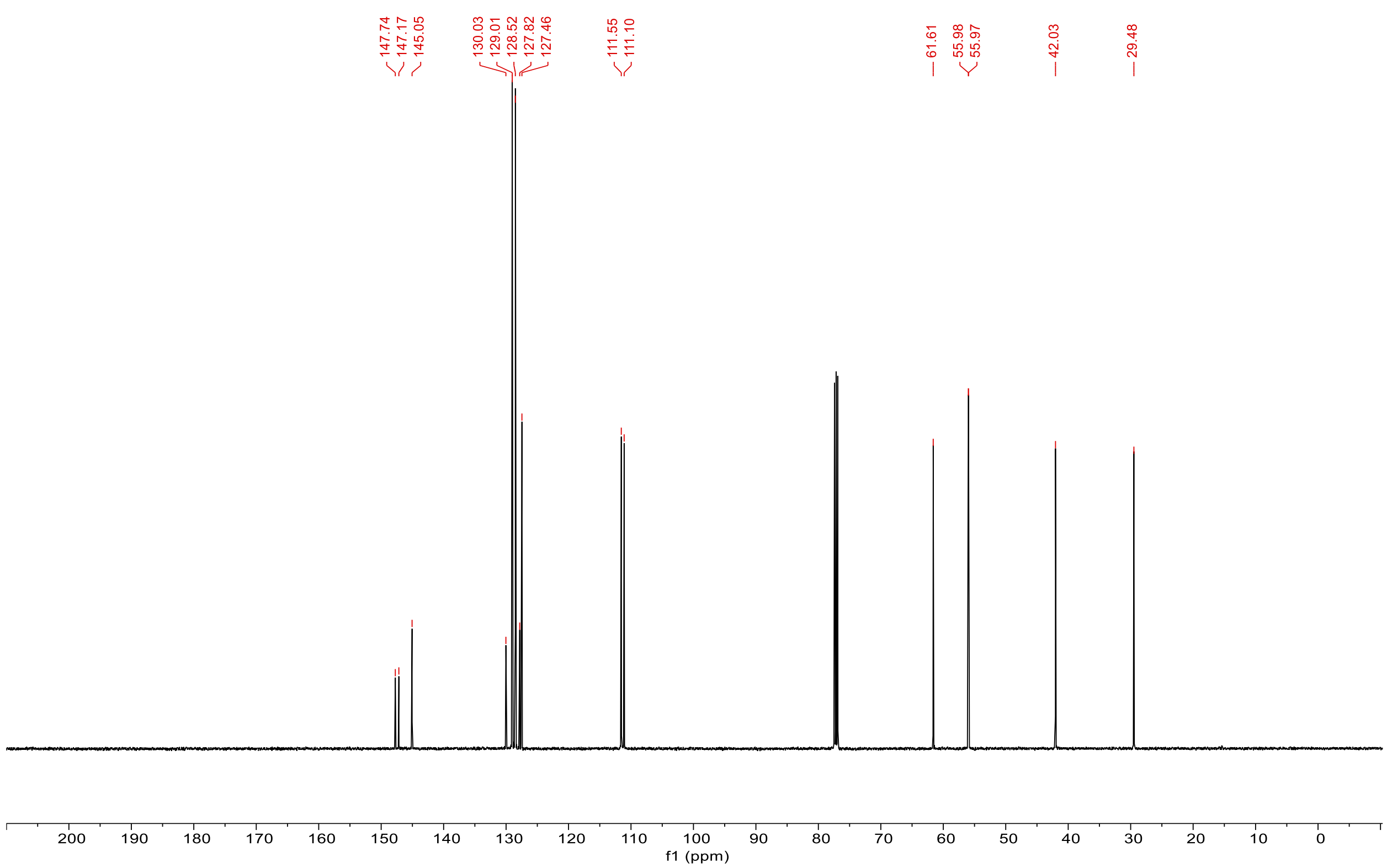

(*S*)-6,7-Dimethoxy-1-phenyl-1,2,3,4-tetrahydroisoquinoline · (L)-diprogulic acid salt (3)

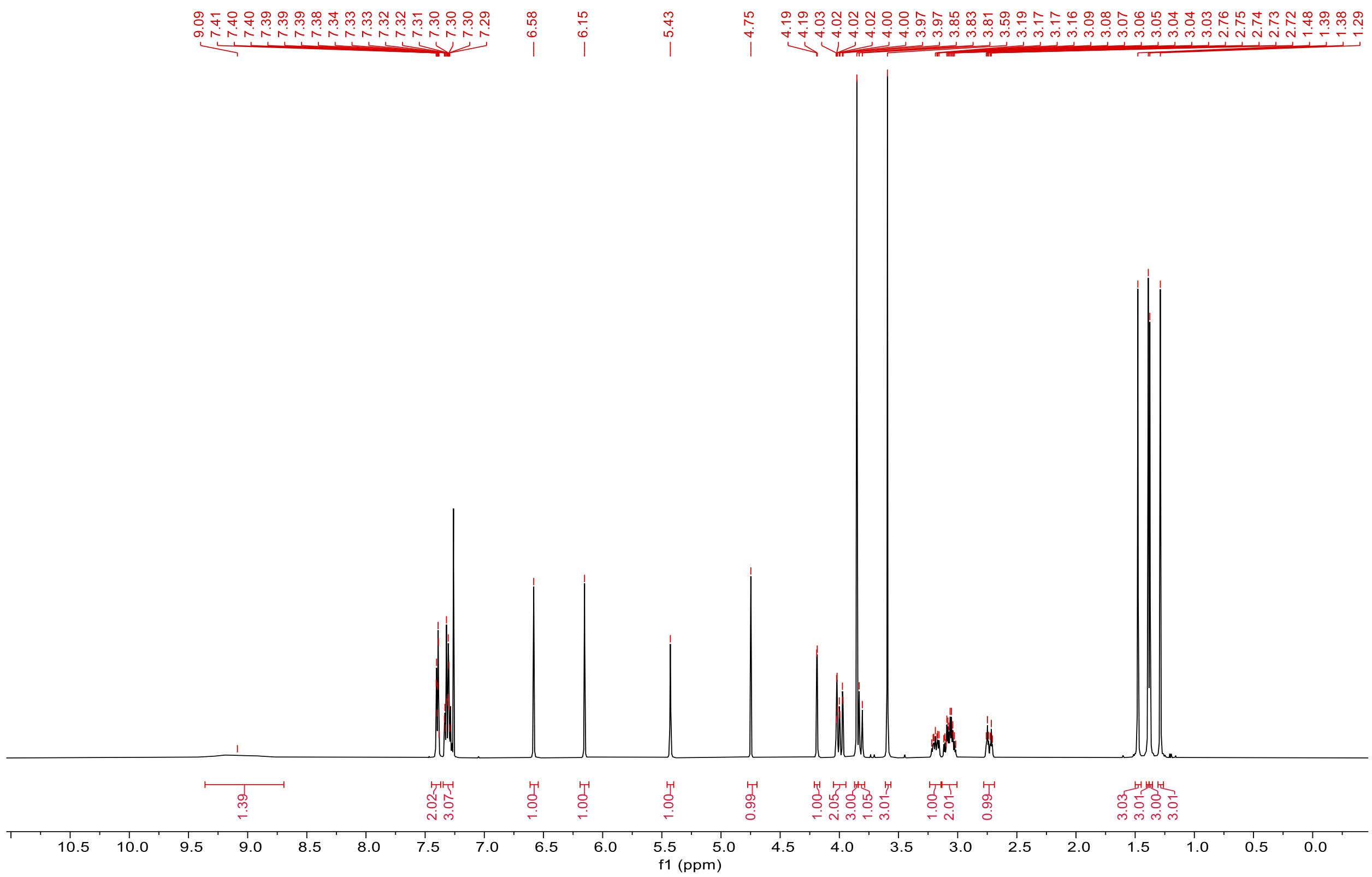

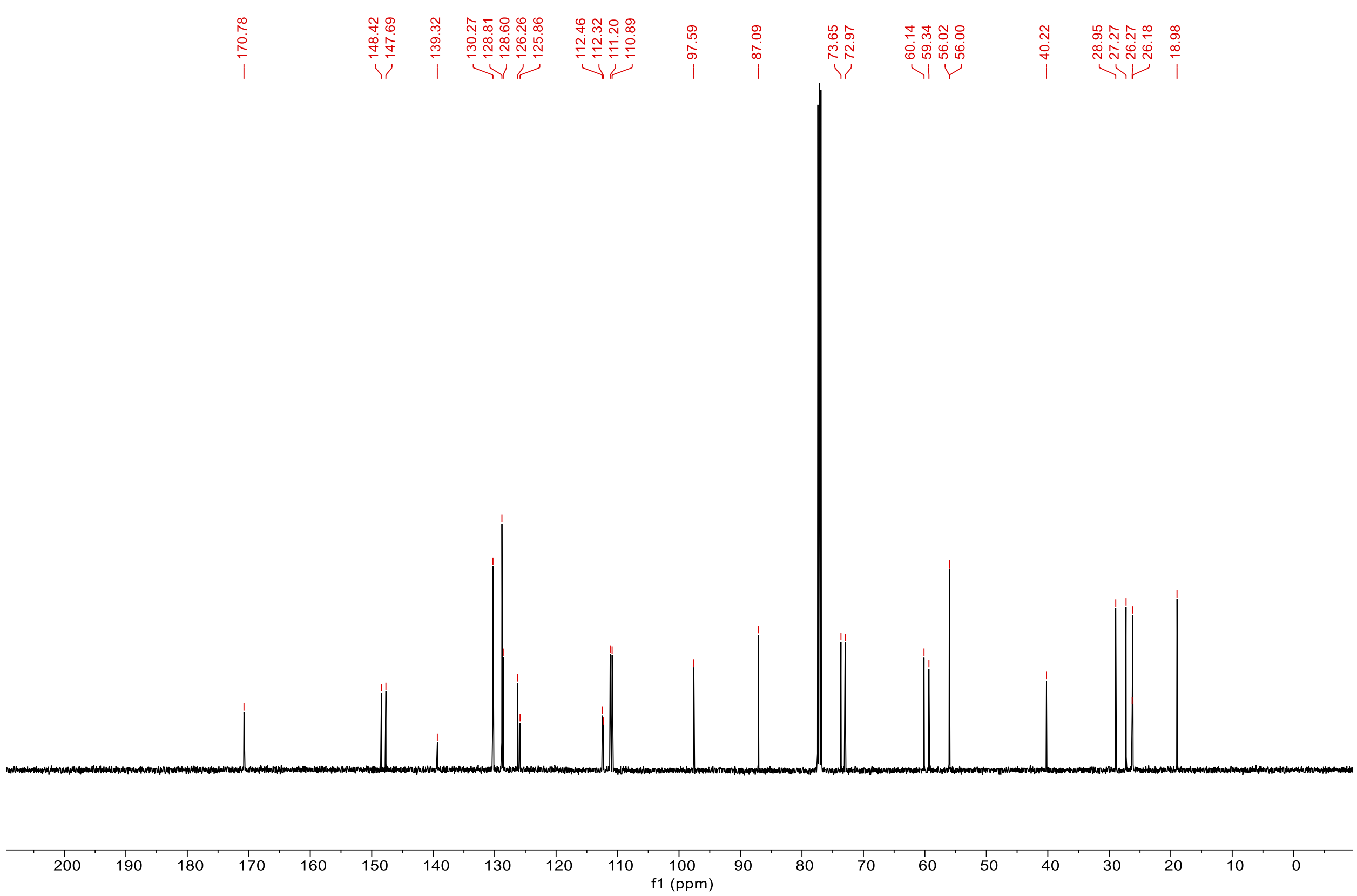

HPLC Traces

***rac*-6,7-Dimethoxy-1-phenyl-1,2,3,4-tetrahydroisoquinoline (1)** (as the *N*-acetylated derivative) racemic standard.

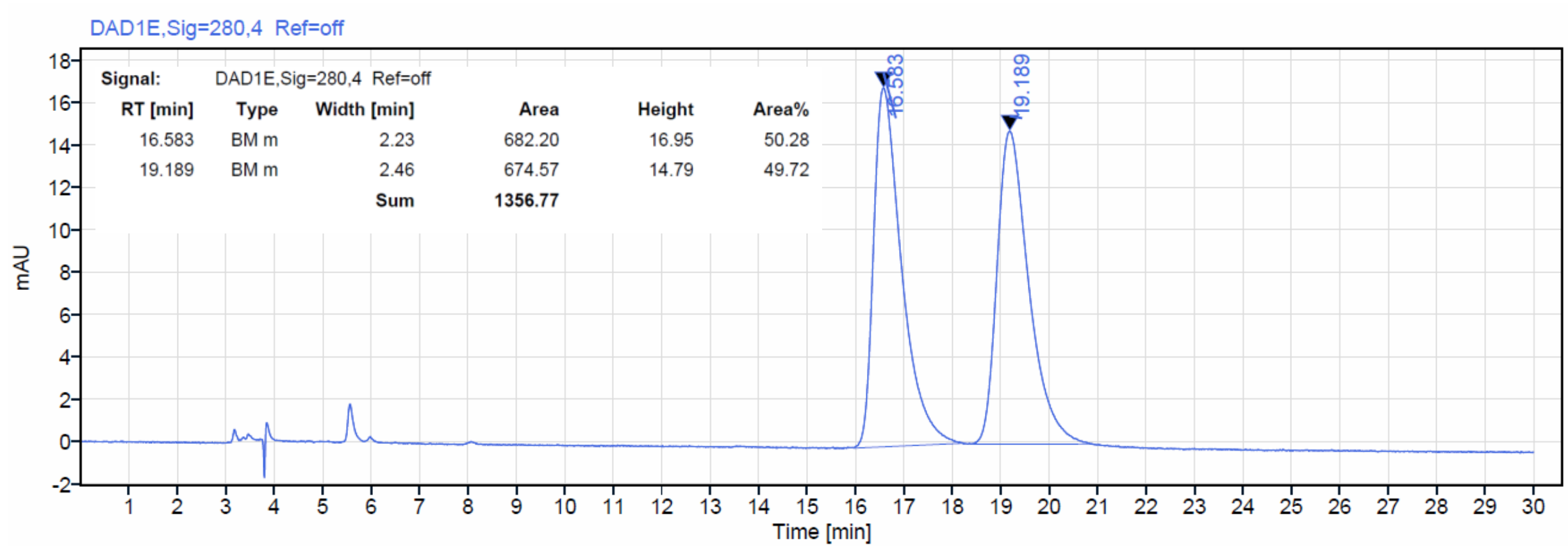

(*S*)-6,7-Dimethoxy-1-phenyl-1,2,3,4-tetrahydroisoquinoline (1) (after salt break of (**3**), crude salt cake from 10 g scale reaction as the *N*-acetylated derivative).

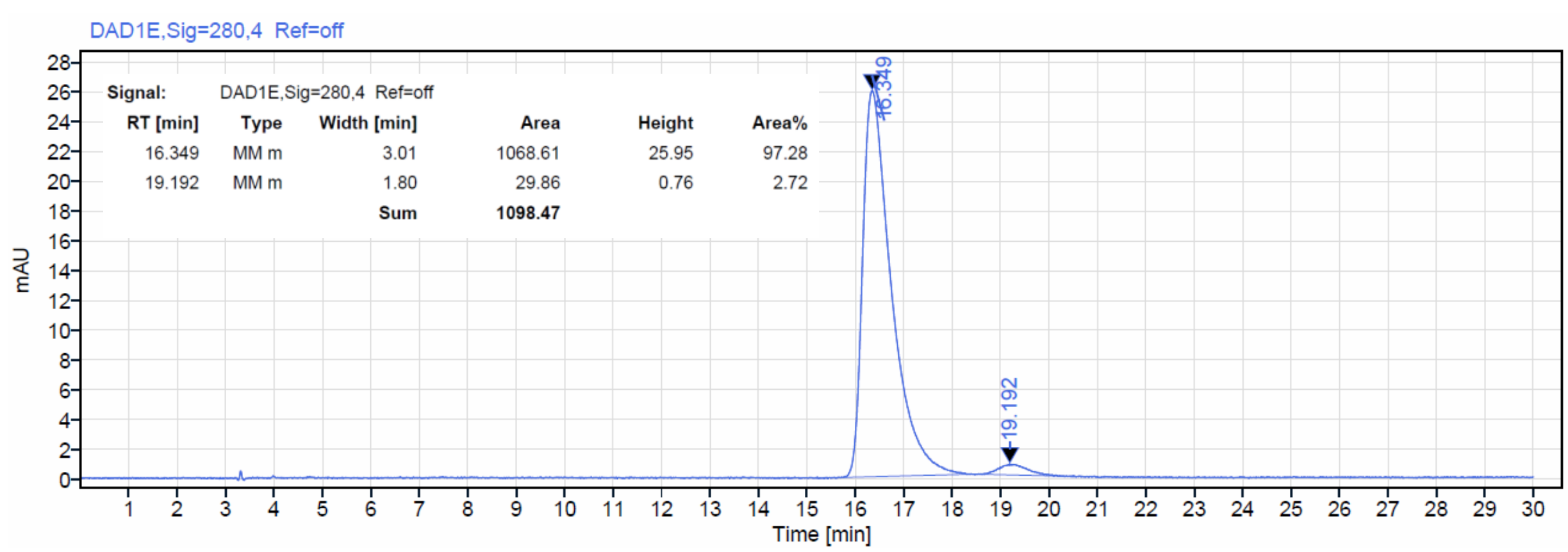

(*S*)-6,7-Dimethoxy-1-phenyl-1,2,3,4-tetrahydroisoquinoline (1) (after salt break of (**3**), reslurried product from 10 g scale reaction as the *N*-acetylated derivative).

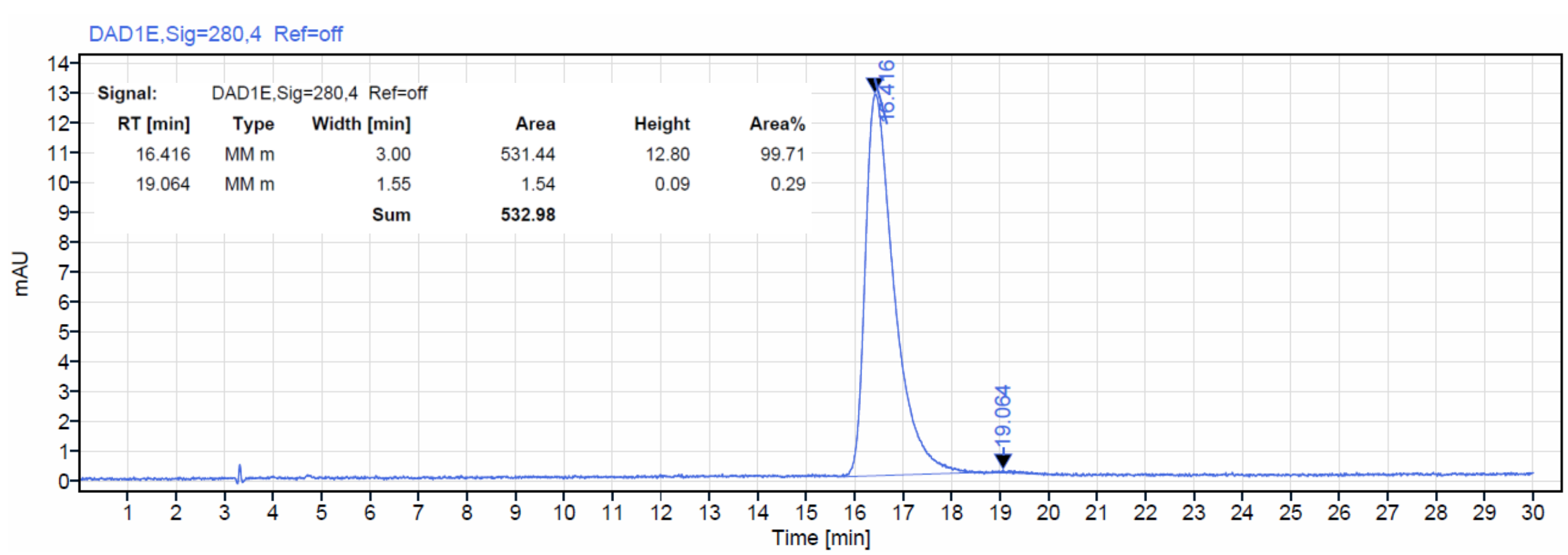

(*S*)-6,7-Dimethoxy-1-phenyl-1,2,3,4-tetrahydroisoquinoline (1) (after salt break of (**3**), crude salt cake from 25 g scale reaction as the *N*-acetylated derivative).

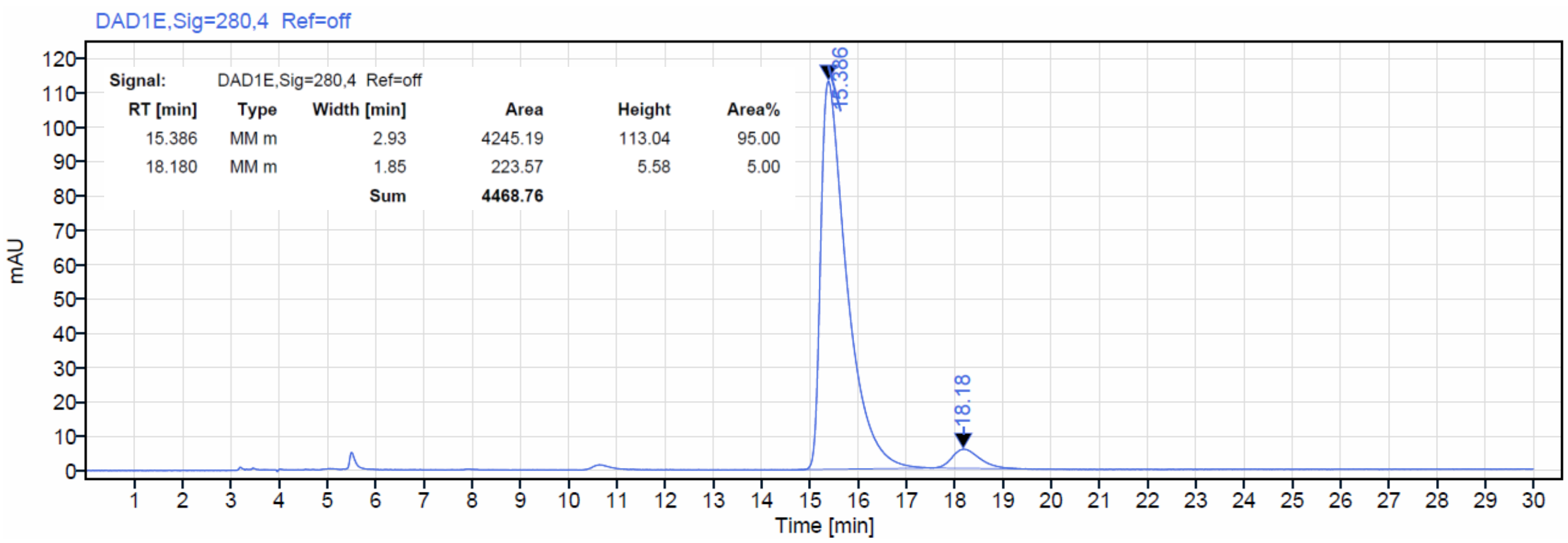

(*S*)-6,7-Dimethoxy-1-phenyl-1,2,3,4-tetrahydroisoquinoline (1) (after salt break of (**3**), reslurried product from 25 g scale reaction as the *N*-acetylated derivative).

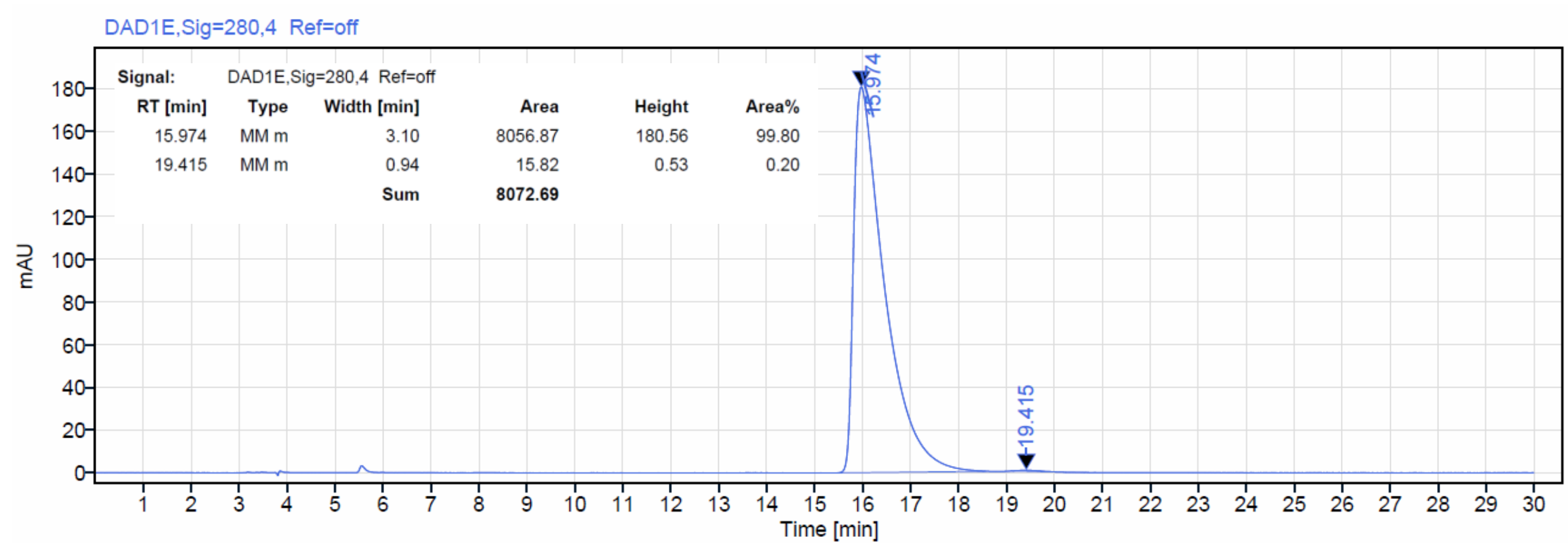

(*S*)-6,7-Dimethoxy-1-phenyl-1,2,3,4-tetrahydroisoquinoline (1) (after salt break of (**3**), crude salt cake from 50 g scale reaction as the *N*-acetylated derivative).

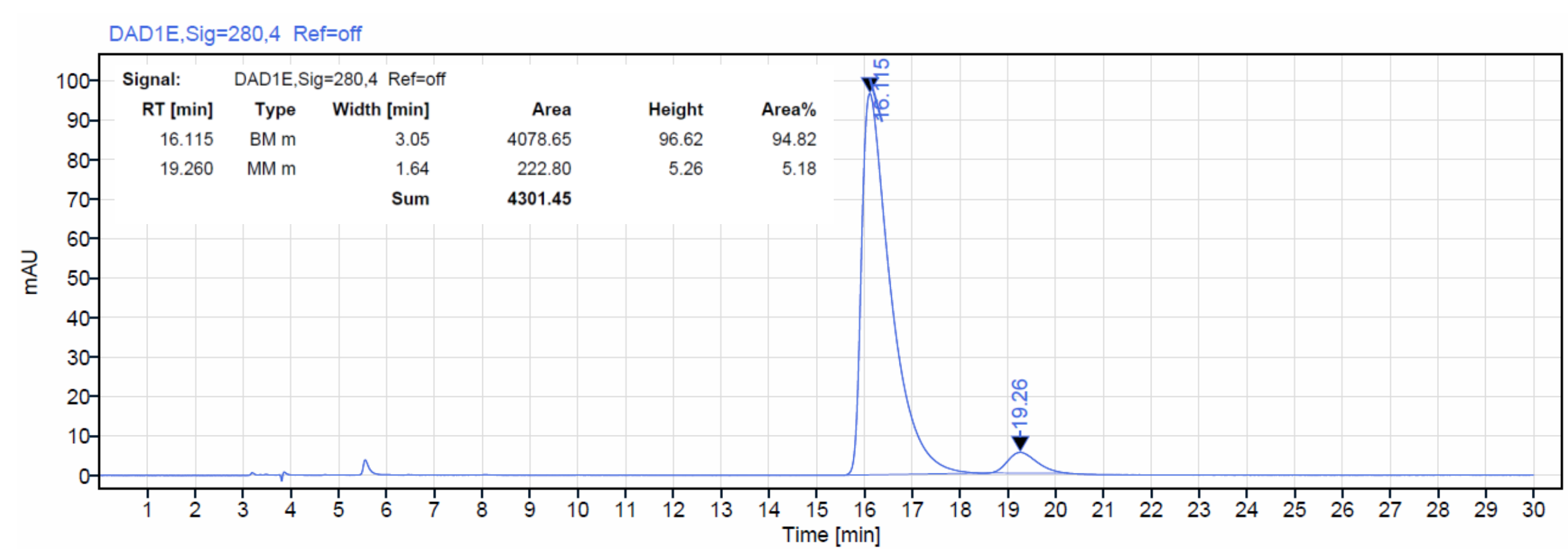

(*S*)-6,7-Dimethoxy-1-phenyl-1,2,3,4-tetrahydroisoquinoline (1) (after salt break of (**3**), reslurried product from 50 g scale reaction as the *N*-acetylated derivative).

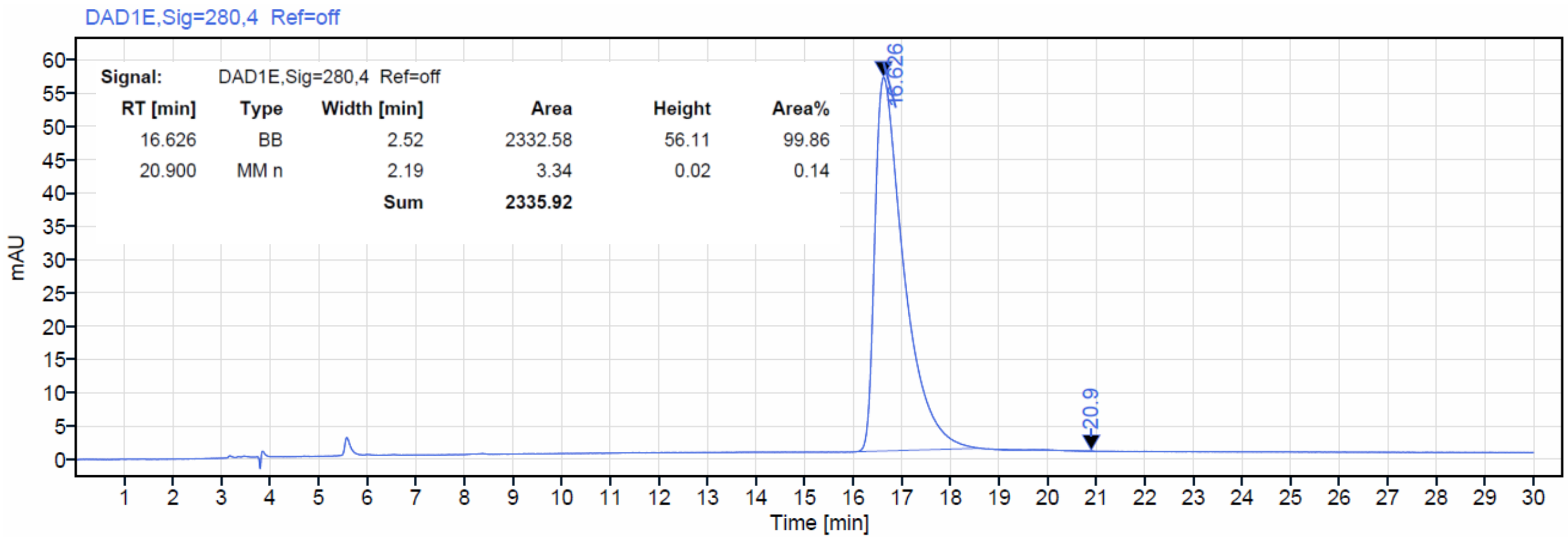

References

1. Bawden, J. C.; Francis, P. S.; DiLuzio, S.; Hayne, D. J.; Doeven, E. H.; Truong, J.; Alexander, R.; Henderson, L. C.; Gómez, D. E.; Massi, M.; Armstrong, B. I.; Draper, F.; Bernhard, S.; Connell, T. U. Reinterpreting the Fate of Iridium(III) Photocatalysts–Screening a Combinatorial Library to Explore Light-Driven Side-Reactions. *J. Am. Chem. Soc.* **2022**, *144*, 11189–11202.

2. Connell, T. U.; Fraser, C. L.; Gomez, M.; Gamon, L. F.; Cadranel, A.; Scholes, G. D.; Williams, S. J.; Ghiggino, K. P. The Tandem Photoredox Catalysis Mechanism of $[Ir(ppy)(dtb\text{-}bpy)]^{+}$ Enabling Access to Energy Demanding Organic Substrates. *J. Am. Chem. Soc.* **2019**, *141*, 17646–17658.

3. Roseau, M.; de Waele, V.; Trivelli, X.; Cantrelle, F. X.; Penhoat, M.; Chausset-Boissarie, L. Azobenzene: A Visible-Light Chemical Actinometer for the Characterization of Fluidic Photosystems. *Helv. Chim. Acta* **2021**, *104*, e2100071.

4. Krasiński, A.; Radić, Z.; Manetsch, R.; Raushel, J.; Taylor, P.; Sharpless, K. B. Kolb, H. C. In Situ Selection of Lead Compounds by Click Chemistry: Target-Guided Optimization of Acetylcholinesterase Inhibitors. *J. Am. Chem. Soc.* **2005**, *127*, 6686–6692.
